# Self-Powered Multimodal Tactile Sensing Enabled by Hybrid Triboelectric and Magnetoelastic Mechanisms

**DOI:** 10.34133/cbsystems.0320

**Published:** 2025-07-02

**Authors:** Xiao Lu, Tianhong Wang, Songyi Zhong, Tianqi Cao, Chenghao Zhou, Long Li, Quan Zhang, Shiwei Tian, Tao Jin, Tao Yue, Shaorong Xie

**Affiliations:** ^1^ School of Computer Engineering and Science, Shanghai University, Shanghai 200444, China.; ^2^ Shanghai Key Laboratory of Intelligent Manufacturing and Robotics, School of Mechatronics Engineering and Automation, Shanghai University, Shanghai 200444, China.; ^3^ School of Electrical Engineering and Automation, Anhui University, Hefei 230601, China.; ^4^ Shanghai Institute of Intelligent Science and Technology, Tongji University, Shanghai 200092, China.; ^5^ School of Future Technology, Shanghai University, Shanghai 200444, China.; ^6^ Shanghai Key Laboratory of Intelligent Connected Vehicle Cybersecurity, Shanghai 200444, China.

## Abstract

Object property perception, as a core component of tactile sensing technology, faces severe challenges due to its inherent complexity and diversity, particularly under the constraints of decoupling difficulty and limited precision. Herein, this paper introduces an innovative approach to object property perception utilizing triboelectric–magnetoelastic sensing. This technology integrates triboelectricity and magnetoelasticity, achieving a self-powered sensing mechanism that requires no external power source for sensing signal generation. Moreover, by deploying a triboelectric array, it comprehensively captures multi-dimensional information of objects. Concurrently, in conjunction with magnetoelastic sensing technology, it provides stable and reliable mechanical information, ensuring that the system can accurately decouple key characteristics of objects, such as material properties, softness, and roughness, even in open environments where temperature, humidity, and mechanical conditions change in real time. Furthermore, by combining deep learning algorithms, it achieves exceptionally high recognition accuracy for object properties (material recognition accuracy: 99%, softness recognition accuracy: 100%, roughness recognition accuracy: 95%). Even in complex scenarios with intertwined multiple properties, the overall recognition accuracy remains consistently above 95%. The multimodal tactile sensing technology proposed in this paper provides robust technical support and theoretical foundation for the intelligent development of robots and the enhancement of real-time tactile perception capabilities.

## Introduction

Tactile sensing technology, an important branch in the field of intelligent robotics, has received extensive attention in recent years [1]. Compared with other sensing methods, the sense of touch is more direct and real, less susceptible to external interference, and can provide a stable and reliable source of information [2]. For robots and intelligent devices, tactile perception enables them to understand the external environment more accurately and enhance the efficiency and naturalness of human–machine interaction [3–5]. For example, during the process of a robot grasping an object, if the robot is equipped with a tactile feedback system, it can adjust the grasping force and method more accurately and avoid damage to the object [6–8].

The primary goal of tactile sensing technology is to simulate the tactile function of human skin and achieve the perception of the properties of objects [9,10]. Different from the singularity of vision and hearing, the multi-dimensionality of the properties of objects, including object shape, size, temperature, material, roughness, softness, etc., poses great challenges for the accurate identification of objects [11]. For the shallow-level information such as the shape, size, and temperature of an object, there are already very mature vision [12–15] and temperature-sensing technologies [16,17] that can easily identify them. However, the material, roughness, and softness of an object have the characteristics of variety, interaction complexity, and fuzzy standards, and these 3 properties are usually integrated with each other, which greatly increases the difficulty of decoupling and accurate identification.

For each of the above 3 properties, there are corresponding sensing mechanisms that can accurately identify them. For example, the material of an object can be identified through a triboelectric array [18,19], the surface texture and roughness of an object can be identified through distributed resistance sensing [20–23], and the softness of an object can be distinguished through piezoelectric and capacitive sensing mechanisms [24–26]. Obviously, a single sensing mechanism cannot decouple these 3 properties simultaneously, which leads researchers to focus on multimodal sensing. However, multimodal sensing is not a simple superposition of sensing mechanisms, and it is necessary to consider the optimal integration mode among sensors and the mutual interference problem between different sensing mechanisms.

Triboelectric sensing based on the principle of triboelectrification and electrostatic induction contains rich object property information. Its electron affinity for different materials and its sensitivity to the micro-structure on the surface of the object endow it with the ability to recognize materials and roughness [27–30]. In addition, triboelectric sensing has the advantages of simple structure, strong expandability, and miniaturization [31–35], enabling it to be integrated with sensors of other mechanisms without interference. Therefore, a multimodal tactile sensing device (MMTSD) based on a triboelectric nanogenerator (TENG) for identifying the properties of objects is a very promising method.

At present, a large number of studies have been devoted to TENG-based multimodal object property recognition devices, including capacitive–triboelectric sensing [36], resistive–triboelectric sensing [37,38], piezoelectric–triboelectric sensing [39], and triboelectric–triboelectric sensing [40]. However, capacitive, resistive, piezoelectric, and triboelectric sensors are greatly affected by environmental factors [41–43]. As a result, their electrical signals will drift considerably in environments with changing temperature and humidity, thus affecting the accuracy of object property identification. Unlike the material and softness of an object, which can respectively eliminate the interference of environmental changes through the ratio of signal normalization between triboelectric arrays and the voltage response time, the identification of object roughness depends on the voltage amplitude information and is greatly affected by force. Therefore, a stable force sensor is needed as the force feedback unit in the TENG-based object property recognition device.

The magnetoelastic effect was recently discovered in a soft materials system [44], which was further coupled with magnetic induction to invent a soft magnetoelastic generator (MEG) as a fundamentally new platform technology for building up human-body-powered soft bioelectronics [45,46]. Thanks to the uniqueness of its signal generation mechanism, it not only is minimally affected by environmental factors [47,48] but also has a highly sensitive force sensing ability [49,50]. In addition, the MEG also has the self-powered ability to convert mechanical energy into electrical energy [51,52], which coincides with the low-power consumption positioning of triboelectric sensing. Therefore, using magnetoelastic sensing as the force feedback unit in the triboelectric-based tactile sensing device is an elegant strategy.

**Fig. 1.**
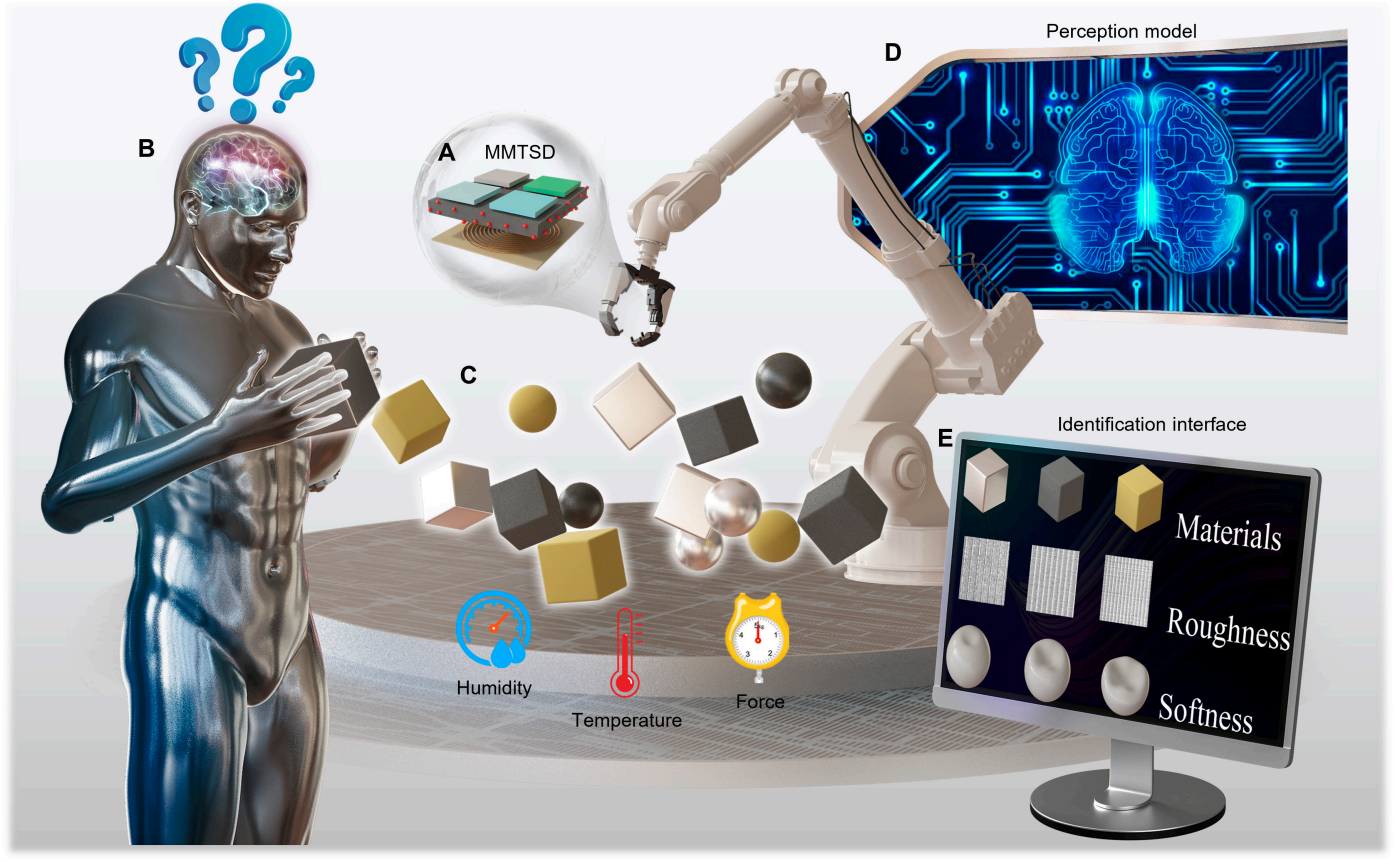
MMTSD-based object property recognition system. (A) MMTSD structure. (B) Anthropomorphic tactile perception. (C) Establishing a dataset in an open environment. (D) Perception model based on CNN. (E) Object properties recognition interface.

Here, we have created an MMTSD based on triboelectric–magnetoelastic (Fig. 1A), which has anthropomorphic tactile sensation (Fig. 1B). This MMTSD effectively interfaces the TENG array with the MEG through silica gel, enabling it to stably perceive the properties of objects within an open environment. To evaluate the efficacy of MMTSD, we used a mechanical claw as the carrier of the MMTSD, carried out grasping tests on 8 material types, 3 softness levels, 4 roughness levels, and 8 objects with intertwined properties and established a comprehensive electrical signal dataset under environmental change (Fig. 1C). Furthermore, we constructed a “material–softness–roughness” perception model based on a lightweight convolutional neural network (CNN) (Fig. 1D), achieving a recognition accuracy rate of more than 95% for materials, softness, and roughness (material recognition accuracy rate: 99.07%, softness recognition accuracy rate: 100%, roughness recognition accuracy rate: 95.56%, and comprehensive property recognition accuracy rate: 95.83%) (Fig. 1E). The MMTSD designed in this study provides a more comprehensive tactile perception ability for robots, improves the intelligence and adaptability of robot operations, and promotes the innovation and development of robots in multiple industries.

## Materials and Methods

### MMTSD’s manufacturing process

**Fig. 2.**
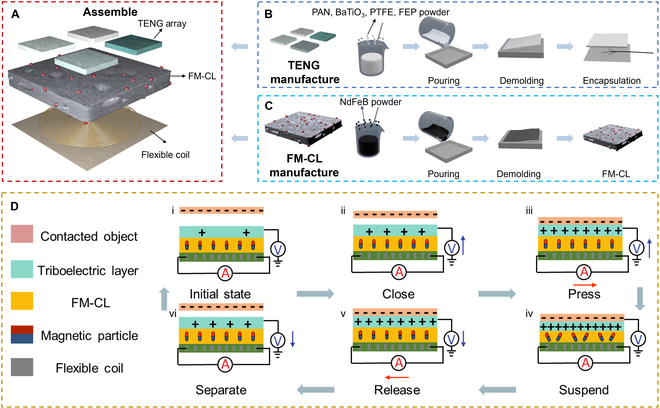
The fabrication and principle of MMTSD. (A) The architecture of MMTSD. (B) The fabrication process of TENG array. (C) The fabrication process of FM-CL. (D) Principle of MMTSD.

The architecture of the MMTSD is illustrated in Fig. 2A. The topmost layer of the device consists of 4 independent silicone-based TENGs arranged in a sensing array configuration. These TENGs are doped with particles that possess different dielectric constants: BaTiO_3_, polyacrylonitrile (PAN), polytetrafluoroethylene (PTFE), and fluorinated ethylene propylene (FEP). This doping strategy enables the achievement of differentiated sensing responses. The intermediate layer features a silicone elastomer impregnated with micromagnets, functioning as the crucial "force-to-magnetic" conversion layer (FM-CL). The bottom layer is engineered as a magnetic induction layer, leveraging printed flexible coil technology to detect and convert changes in the magnetic field into electrical signals.

For the fabrication of the TENGs, 4 types of particles with distinct dielectric constants—BaTiO_3_, PAN, PTFE, and FEP—are incorporated into a silicone matrix composed of Ecoflex 00-30 Parts A and B, which are mixed in a 1:1 ratio. This mixture serves as the composite material for the sensing array. Subsequently, the mixture is stirred thoroughly with a glass rod for 5 min to ensure homogeneity. To remove the bubbles generated during the mixing process, a vacuum pump is utilized, thus maintaining the material’s uniformity. Following this, the mixture is injected into a mold, and after demolding, the triboelectric sensing element of the MMTSD is successfully fabricated (Fig. 2B).

The preparation of the MEG entails a more elaborate procedure. First, neodymium-iron-boron (NdFeB) micromagnets, which account for 75% of the total mass, are combined with the Ecoflex 00-30 Parts A and B mixture. To create a microporous structure within the magnetoelastic film, the mixture is stirred for 10 min, intentionally introducing small bubbles. The uniformly blended slurry is then injected into a custom 3D-printed mold and undergoes drying, heating, and curing processes at 75 °C for 3 h in an oven (Fig. 2C). Once cured, the magnetoelastic film is magnetized using a MAG series high-voltage capacitive magnetizing power supply at 226 V, thereby enhancing its magnetic response characteristics. Finally, a protective layer of silicone rubber, approximately 1 mm in thickness, is applied to encapsulate both the magnetoelastic film and the flexible metal coil, protecting the internal components and enhancing the overall durability of the device. An optical photograph of the MMTSD is presented in Fig. S1.

### Principle of MMTSD

The signal generation mechanism of the MMTSD is illustrated in Fig. 2D. In phase (i), upon contact with an external object, positive charges accumulate on the object due to the triboelectric effect. Meanwhile, because of the relatively large initial distance, only a negligible amount of negative charge is acquired by the silicone composite film.

In phase (ii), as the external object approaches, the electrostatic induction effect causes a change in the potential difference between the silicone composite film and the ground. This potential difference drives the migration of free electrons from the ground to the film, thus initiating a circuit current. During phase (iii), when the external object applies contact pressure, the MMTSD deforms. As the distance between the object and the film decreases, a greater number of free electrons are attracted to the silicone composite film, which notably enhances the electrical signal. Simultaneously, the compressive force deforms the wavy chain structure of the micromagnets within the FM-CL, leading to a reduction in the surface magnetic flux density. According to Faraday’s law of electromagnetic induction, the flexible printed coil accurately detects this variation in the magnetic field, resulting in a corresponding current output.

When the external object makes full contact with the MMTSD in phase (iv), the triboelectric charge reaches saturation, and the electron flow between the ground and the silicone composite film achieves dynamic equilibrium. Moreover, the magnetic flux density on the surface of the FM-CL drops to its lowest value, preventing the generation of induced current. Subsequently, in phase (v), as the external pressure is released, the silicone composite film gradually reverts to its original shape. This causes the free electrons to flow in the opposite direction, from the film back to the ground, generating a reverse current. Concurrently, the wavy chain structure of the micromagnets in the FM-CL also gradually recovers, inducing a reverse current in the flexible metal coil and restoring the magnetic flux density to its initial state.

Finally, in phase (vi), during the separation of the external object from the MMTSD, the decreasing proximity reduces the number of electrons on the silicone composite film, once again triggering a current in the same direction as during the approach phase. The intensity of this current gradually increases with the growing distance, ultimately returning the system to the stable state observed in phase (i). This process demonstrates the MMTSD’s ability to achieve efficient energy conversion and signal output, which is enabled by the coordinated interaction of the triboelectric and electromagnetic induction effects within the dynamic contact–separation cycle.

### Characterization and measurement

In this study, standardized test signals were generated utilizing the LIMNOT stepper motor, which is capable of achieving precise position output through the pre-setting of various path curves. The stepper motor’s frontend design incorporates a versatile structure that accommodates objects of diverse material shapes, facilitating collision tests on the MMTSD. For the evaluation of electrical characteristics, the Keithley 6514 high-precision electrical measurement system and the Arduino data acquisition platform were employed, directly displaying measurement results on an oscilloscope to ensure both the intuitiveness and accuracy of the data.

To delve deeper into the impact of temperature and humidity on MMTSD’s performance, this study incorporated a heating plate and humidifier as environmental control devices. A temperature and humidity detector were utilized to capture real-time temperature and humidity data of the environment. In the context of an actual application test scenario, the MMTSD was securely mounted on the clamping module of the Zhixing adaptive servo electric gripper (CTAG series). The Arduino and National Instruments (NI) data acquisition card module were leveraged to gather voltage and current data in real time, while a notebook computer was used for data storage and visualization processing. This comprehensive approach enabled a rigorous evaluation of MMTSD’s performance under complex environmental and dynamic conditions.

### Object property perception system

A set of serial port data acquisition and display software has been developed utilizing Python and the PyQt5 framework. This software employs multi-threading technology to achieve real-time reading and processing of serial port data, thereby ensuring prompt updates and intuitive data display. The collected data are visualized as a real-time waveform within a designated window area through matplotlib plotting. After the sensor data are efficiently recognized and processed by the integrated algorithm, accurate judgment results are promptly presented and displayed via a user-friendly interface. The software interface is designed with simplicity and clarity in mind, incorporating multiple functional buttons such as those for initiating/halting data acquisition, displaying test results, real-time waveform visualization, and clearing display content. This comprehensive feature set provides users with a convenient and streamlined operational experience.

## Results

### Performance of MMTSD

The structural parameters of the FM-CL, including its thickness, the doping concentration of magnetic powder particles, and the area, markedly impact the magnetoelastic sensing performance. Based on the fundamental principles of the MEG, a direct relationship exists between the number of coil turns and the output performance of the MEG. An analysis of their influence on MEG performance is presented in Note S1 and Fig. S2. Similarly, Note S2 and Fig. S3 detail the mechanisms through which the area of the triboelectric layer and the microstructural proportion affect TENG performance.

**Fig. 3.**
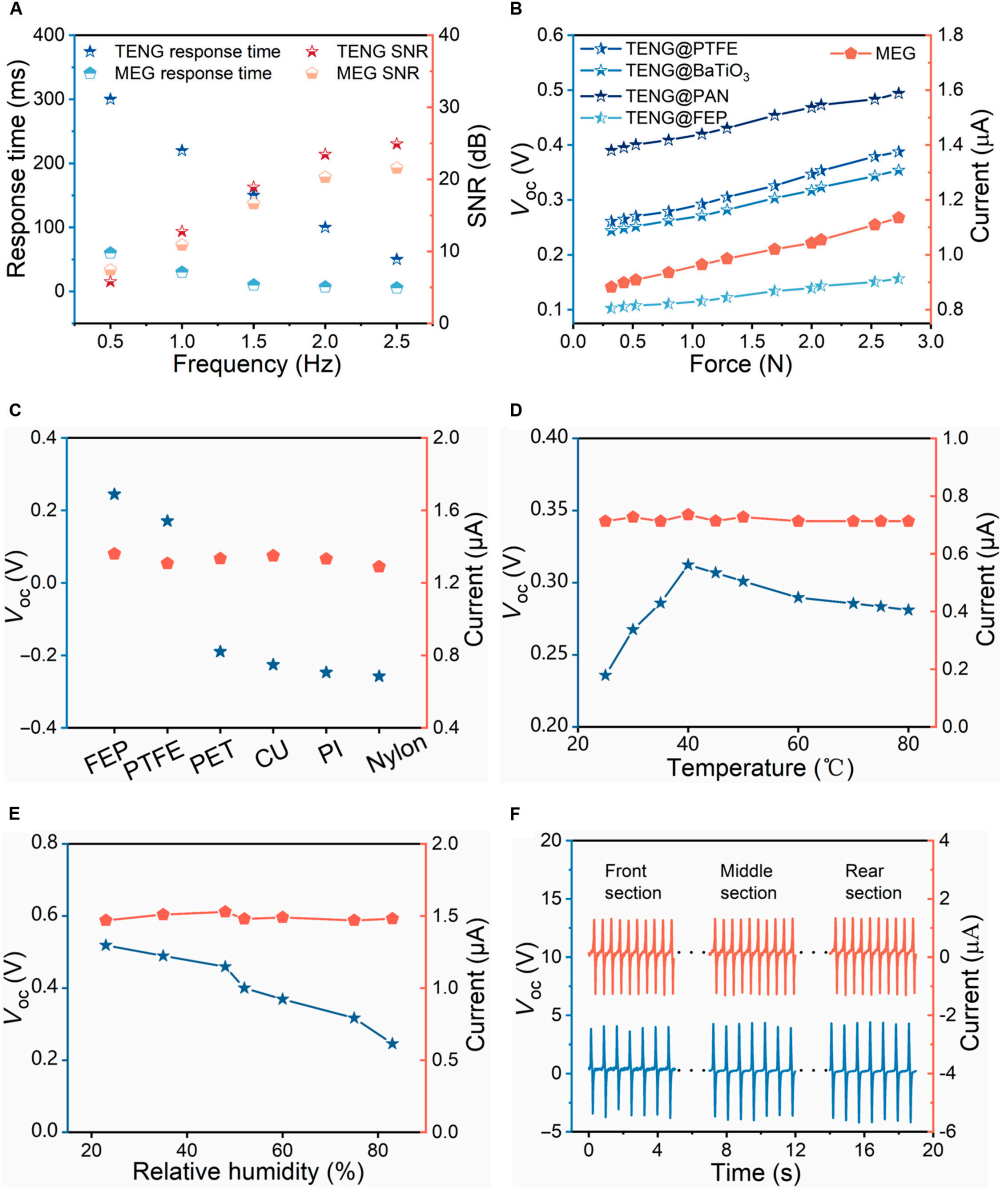
Performance of MMTSD. (A) Frequency response of MMTSD. (B) Force response curve of MMTSD. (C) The influence of the material of the touched object on the electrical signal of MMTSD. (D and E) The influence of temperature and humidity on MMTSD. (F) The cyclic stability of MMTSD.

To evaluate the frequency response of the MMTSD, this paper applied contact forces of varying frequencies under constant pressure to the device. The experimental results are presented in Fig. S4. Fig. 3A illustrates the performance of both the MEG and the TENG in terms of response time and signal-to-noise ratio (SNR). The experimental data demonstrate that as the applied frequency increases from 0.5 to 2.5 Hz, the response time of the TENG decreases from 300 to 50 ms, while the SNR increases from 5.79 to 24.92 dB. Correspondingly, the MEG also shows a reduction in response time, from 60 to 5.5 ms, with an increase in the SNR from 7.43 to 21.57 dB.

To further investigate the pressure-sensing capabilities of the MMTSD, a tensile testing machine was used to apply precise forces, and the resulting force–electrical output relationship curve was plotted (Fig. 3B). The results show that as the pressure applied to the MMTSD increases from 0 to 3 N, the current signal of the MEG exhibits a strong linear growth trend, reaching a value of 1.2 μA. Simultaneously, the voltage signals generated by the 4 TENGs display similar linear characteristics, as shown in Table S1, confirming their effectiveness in pressure-sensing applications.

Based on the theoretical foundations of the TENG and MEG, it is clear that the inherent disparities in electron affinity among different materials directly affect the quantity of triboelectrically polarized charges, thereby regulating the output signal of the TENG. In contrast, the signal of the MEG is largely insensitive to material variations, as it mainly depends on changes in magnetic flux. This unique characteristic provides the rationale for our subsequent efforts to decouple the properties of objects.

To verify this hypothesis, we performed experiments under consistent conditions, measuring the signal outputs from 7 distinct materials. As expected, the TENG signal showed substantial fluctuations with material changes, while the MEG signal remained relatively constant, as illustrated in Fig. 3C. In a dynamic open-space environment, where numerous environmental factors are present, temperature and humidity are key determinants of sensor performance. Therefore, we focused on evaluating the performance of the MMTSD under varying temperature and humidity conditions.

Fig. 3D elucidates the effect of temperature, indicating that the MEG signal demonstrates strong temperature stability. Conversely, the TENG signal initially increases and then decreases as the temperature rises, reaching a peak of 0.3 V at 40 °C. Similarly, Fig. 3E depicts the impact of humidity, with the MEG signal maintaining stability, whereas the TENG signal exhibits a downward trend. Specifically, at 80% relative humidity, the TENG voltage decreases by approximately 52.6%. This phenomenon can be attributed to the differences in environmental adaptability between the TENG and MEG. The magnetoelastic sensing mechanism, owing to its distinctive sensing principle, has remarkable advantages in waterproof performance. In contrast, the triboelectric sensing mechanism relies heavily on the surface triboelectrification effect, making the TENG highly sensitive to environmental humidity.

Despite the inhibitory effect of humidity on the output performance of triboelectric sensing, it does not undermine its viability in future applications. First, even in an extreme environment with a relative humidity of 100%, the TENG can still generate discernible voltage signals. Second, during the dataset construction process, to fully account for the impact of environmental humidity on the performance of the MMTSD, this paper collected multimodal electrical signals across multiple humidity gradients. Additionally, during the data processing stage, the collected data were standardized to further mitigate the influence of humidity.

Furthermore, we analyzed the power supply performance of both the TENG and MEG components, investigating the relationships among voltage, current, power, and load. The data supporting this analysis are presented in Note S3 and Fig. S5. Finally, cyclic stability is a critical indicator for evaluating sensor performance. To assess this, we used a linear motor to subject the MMTSD to approximately 5,000 consecutive impact tests, recording the voltage and current data from its front, middle, and rear sections, as shown in Fig. 3F (detailed data are presented in Fig. S6). Remarkably, throughout the testing period, neither the amplitude of the TENG voltage nor the MEG current showed noticeable changes, demonstrating the excellent cyclic stability of the MMTSD.

### Object property analysis based on MMTSD electrical signal

In the domain of robotic tactile perception, the perception of object properties is a crucial element that boosts the adaptability and intelligence of robots operating in complex and dynamic environments. This, in turn, broadens their range of potential applications across multiple fields. The fundamental properties of objects, such as material composition, softness, and roughness, are intricately interconnected, presenting a considerable challenge in terms of the technical difficulties involved in separating these properties and achieving precise identification. Triboelectric sensing is an emerging tactile perception technology that exploits the difference in electron affinity between objects to generate electrical signals rich in information about the surface characteristics of these objects. These signals form the basis for accurately discerning material type, softness, and roughness.

Object material identification: The unique charge distribution patterns on the surfaces of different materials serve as the foundation for the effectiveness of triboelectric sensing in material identification. By capturing and analyzing the specific magnitudes of these charges, this technology can achieve high accuracy in differentiating between materials. The key lies in quantifying and distinguishing the differential peak voltages resulting from the varying amounts of charge on material surfaces. However, relying solely on a single triboelectric sensor is limited by fluctuations in environmental factors, including contact force, temperature, and humidity. To overcome these limitations, the integration of triboelectric arrays has proven to be a crucial strategy, enhancing both the robustness and precision of identification. In this setup, TENGs with different electron affinities generate distinct peak voltage signals upon contact with an object. The relative proportions between these peaks remain relatively constant, enabling efficient and stable material identification in uncontrolled environments [18].

Object softness identification: The differentiation between soft and hard objects is based on the varying degrees of surface deformation under a standardized external force. Softer objects deform more drastically, leading to longer contact durations. This, in turn, results in more pronounced variations in the electrical signal of the MMTSD, allowing for the effective discrimination of object softness [40].

Object Roughness Identification: The microscopic topography of an object’s surface is central to roughness identification. Smooth surfaces are characterized by flatness, while rough surfaces have numerous grooves. Whereas humans typically rely on tactile sliding motions to perceive surface irregularities, triboelectric sensing employs a pressing approach. Smooth surfaces have a larger contact area, which induces greater charge polarization and generates higher voltage peaks, providing the basis for roughness identification. However, the sensitivity of triboelectric signals to external force variations limits their direct and widespread application. To address this, integrating the MEG as a force compensation unit provides stable contact force information. The combination of MEG-derived force information with triboelectric signals effectively decouples and accurately identifies the roughness of object surfaces [19].

**Fig. 4.**
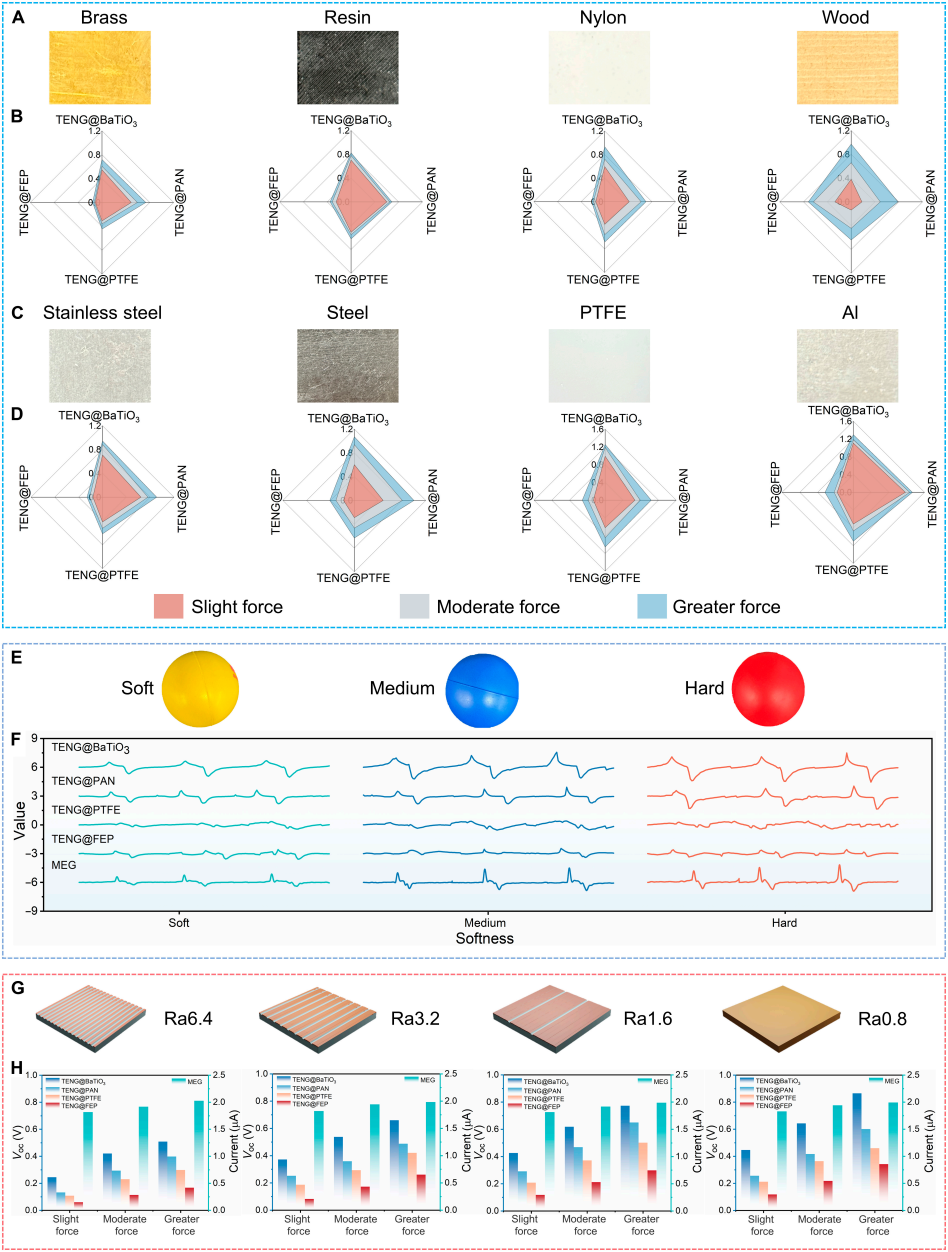
MMTSD electrical signal analysis for properties of objects. (A to D) Optical photos and TENG voltage peak radar maps corresponding to 8 different material objects. (E) Three different softness levels of balls and (F) their corresponding MMTSD electrical signal diagrams. (G) Four different roughness steel blocks and (H) their corresponding MMTSD peak histograms.

During the investigation of how the electrical signal analysis of MMTSD disentangles object properties, the outstanding compatibility features of MMTSD were utilized to integrate it into a robot. Specifically, using a double-clamp mechanical claw as a practical application, MMTSD was incorporated into the claw’s structure, thus creating an intelligent mechanical manipulation platform capable of recognizing object properties, as shown in Fig. S7. To validate the system’s performance, an experiment was designed. By adjusting the grasping operations with 3 different forces, 8 different materials of objects were tested, as illustrated in Fig. 4A and C. The acquired electrical signal waveforms are presented in Fig. S8. These waveforms were then processed to extract key peak voltage information.

When these peak voltage data were transformed into radar charts (Fig. 4B and D), a notable phenomenon was observed: even when different forces were applied to the same material, although the peak voltage increased with increasing force, the geometric shape enclosed by the voltage peaks generated by the TENG array remained unchanged. This observation highlights the stability and uniqueness of triboelectric signals in material identification. Additionally, it was found that different materials produced different voltage peak shapes under the same test conditions. These can be regarded as an inherent “electrical fingerprint” or code specific to each material, showing a clear one-to-one correspondence. To visually emphasize this characteristic, the data were normalized and presented as a histogram (Fig. S9 and Table S2). The analysis revealed that the peak voltages measured under different forces had similar numerical values after normalization, firmly establishing the TENG array’s strong resistance to force variations during the recognition process.

To evaluate the electrical signal characteristics of MMTSD when interacting with objects of different softness, this paper selected 3 small balls with varying stiffness as test samples for the grasping experiment. The experimental setup is shown in Fig. 4E. During the experiment, 3 predefined grasping forces were consistently applied for testing, and the corresponding electrical signal output of MMTSD was recorded. The results are presented in Fig. 4F. The analysis indicated that as the softness of the object increased, the response time of the electrical signal tended to lengthen, accompanied by a corresponding decrease in voltage amplitude. The quantitative details of this finding are provided in Table S3.

Furthermore, in the exploration of the ability to distinguish object roughness, steel blocks with 4 surface roughness values (Ra0.8, Ra1.6, Ra3.2, and Ra6.4) were introduced as test subjects, as shown in Fig. 4G. The mechanical gripper equipped with MMTSD was used to perform the grasping operations, and the real-time electrical signals generated by MMTSD were recorded. The data are presented in Fig. S10. Preliminary analysis showed that relying solely on triboelectric signals under different grasping forces made it difficult to accurately distinguish object roughness, as demonstrated in Note S4 and Fig. S11. However, by integrating the force feedback information provided by MEG, the accuracy of roughness recognition was notably improved. To visually demonstrate this improvement, a histogram was plotted showing the electrical signal responses of MMTSD to objects with the same roughness under different forces (Fig. 4H). This figure clearly showed that for objects with the same roughness, as the grasping force increased, the amplitude of the electrical signal increased. Notably, the ratio of the electrical signal amplitude remained the same under the same roughness condition, while showing appreciable differences between different roughnesses. This finding validated that incorporating the force feedback mechanism substantially enhanced the effectiveness and precision of MMTSD in roughness recognition tasks. A comparison with other studies on object property identification, as shown in Table S4, fully demonstrated the superiority of this work in terms of recognition categories and accuracy.

**Fig. 5.**
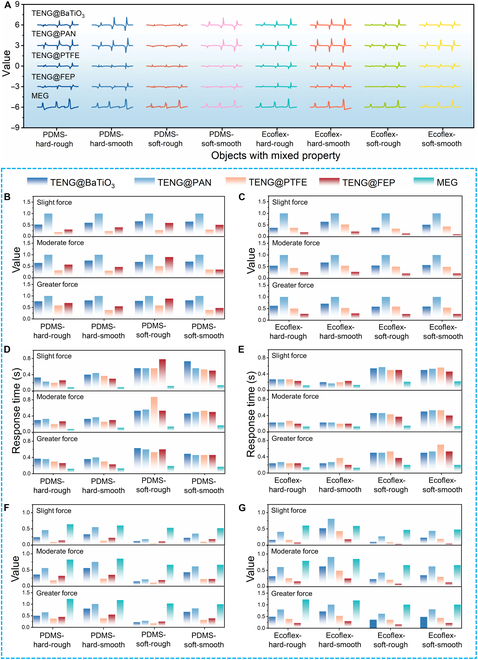
MMTSD electrical signal analysis for the 8 objects with intertwined properties under different forces. (A) MMTSD electrical signals of the 8 objects with intertwined properties. (B and C) Standardization data of TENG voltage peak values for the 8 objects with intertwined properties. (D and E) MMTSD response time for the 8 objects with intertwined properties. (F and G) The peak value of MMTSD’s electrical signal for the 8 objects with intertwined properties.

To thoroughly evaluate the ability of MMTSD to analyze and distinguish object characteristics, 8 specimens with various combinations of properties were created, where material composition, softness, and roughness were intricately intertwined. These specimens were systematically classified according to the “material–softness–roughness” paradigm, including “PDMS–hard–rough”, “PDMS–hard–smooth”, “PDMS–soft–rough”, “PDMS–soft–smooth”, “Ecoflex–hard–rough”, “Ecoflex–hard–smooth”, “Ecoflex–soft–rough”, and “Ecoflex–soft–smooth”. The method for parameter adjustment is described in Note S5 and Fig. S12. Using a mechanical gripper equipped with MMTSD, these objects were manipulated, and the corresponding electrical signatures were recorded in Fig. 5A.

A radar chart, shown in Fig. S13, illustrated the peak triboelectric signals from Ecoflex and PDMS-based materials. Notably, the shapes formed by the peak voltage signals of TENG in PDMS were very similar, distinguishing them from those of Ecoflex materials. Fig. 5B and C shows the standardized peak voltage data of TENG based on PDMS and Ecoflex materials, respectively, providing a more intuitive comparison of the differences between the 2 materials. Furthermore, the feature values of MMTSD electrical signals were extracted (detailed data can be found in Tables S5 to S7). Fig. 5D and E presents a histogram analysis of the electrical signal response times. The data revealed a consistent trend: When MMTSD interacted with PDMS/Ecoflex materials of the same hardness, the response time remained relatively constant. In contrast, when interacting with softer PDMS/Ecoflex materials, the response time was notably longer. Fig. 5F and G shows the histogram analysis of the peak values of MMTSD electrical signals. Interestingly, due to the accurate force feedback of MEG, the ratios of peak electrical signals of Ecoflex/PDMS materials with the same softness but different roughnesses differed markedly. Specifically, the voltage signal of MMTSD increased with both roughness and force, while the current signal only increased with force.

### Object property recognition model

In the previous chapter, an extensive analysis was conducted on the object property recognition mechanism based on the MMTSD. Although the unique characteristics of MMTSD electrical signals can act as an initial means for property discrimination, the complex and multi-faceted nature of these properties often renders traditional manual feature extraction methods inadequate for capturing their comprehensive attributes, such as material type, softness, and roughness. Therefore, to achieve a more precise and comprehensive recognition of object properties, this paper introduces a deep learning algorithm. This algorithm is designed to automatically extract in-depth features and subsequently perform efficient and accurate classification of material types, softness levels, and roughness grades.

**Fig. 6.**
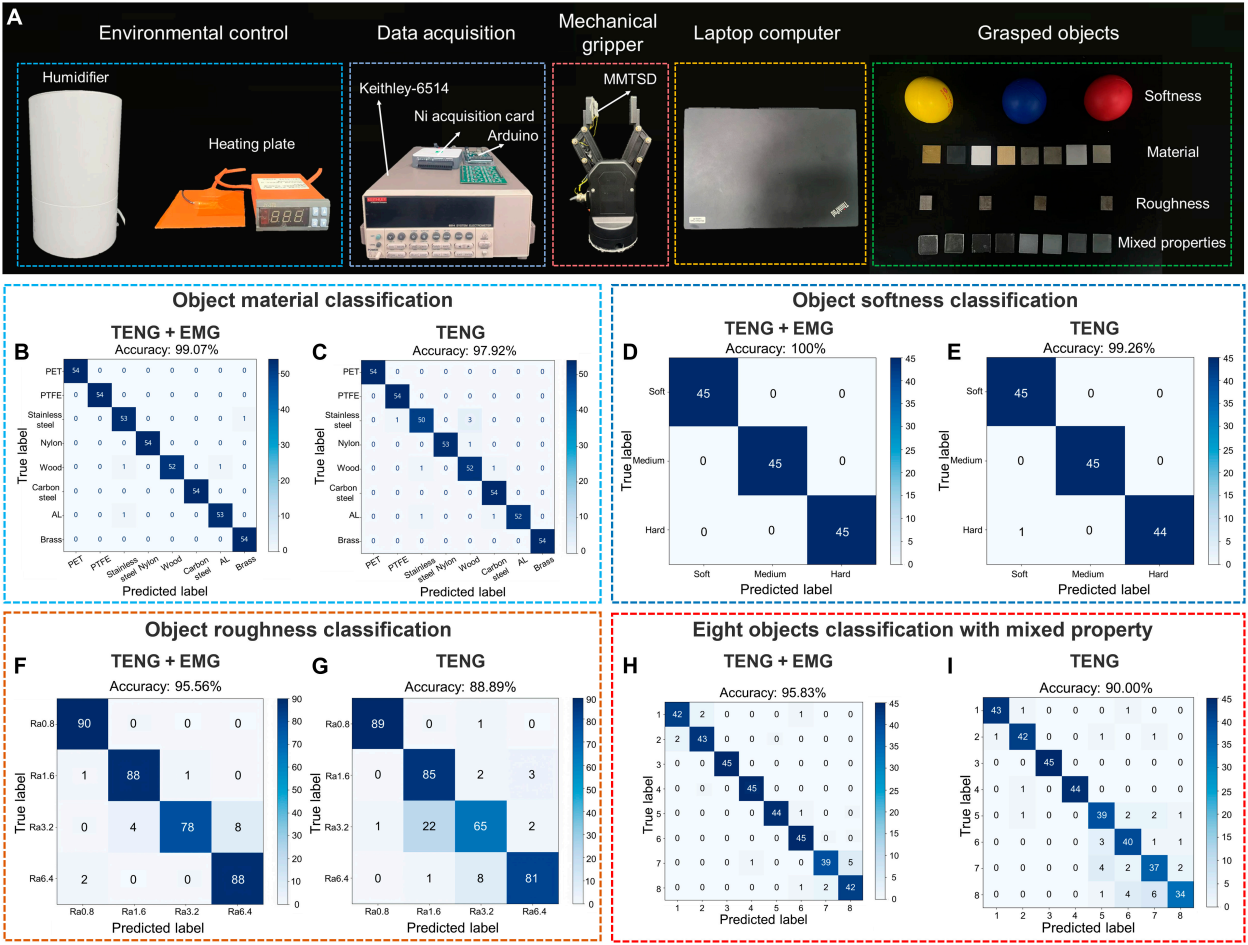
Dataset collection platform and performance of the object property recognition model. (A) Dataset collection platform. (B and C) Confusion matrices trained on different datasets in material classification tasks. (D and E) Confusion matrices trained on different datasets in softness classification tasks. (F and G) Confusion matrices trained on different datasets in roughness classification tasks. (H and I) Confusion matrices trained on different datasets in the 8 objects with intertwined properties classification tasks.

Here, an object property recognition model (OPRM) based on a simplified CNN has been developed, and its framework is illustrated in Fig. S14. To ensure the robustness of the OPRM in real-world application scenarios, a training dataset was collected under various conditions of temperature, humidity, and mechanical factors. The configuration of the adopted test platform is shown in Fig. 6A. This platform is located within a sealed enclosure constructed from acrylic and is equipped with the following instruments:

Mechanical gripper: The mechanical gripper, integrated with MMTSD, is capable of sensing object properties. Parameters such as grasping force and the number of grasping times are controlled by a specialized mechanical gripper driver program.

Data acquisition equipment: This includes a Keithley-6514, an Ni data acquisition card, and an Arduino controller. Specifically, the electrometer and Ni data acquisition card are synchronized to collect current data from the MEG, while the Arduino is responsible for acquiring voltage data from the triboelectric array.

Laptop computer: Equipped with data-reading software, this device enables the real-time storage and retrieval of both current and voltage data.

Humidifier: This device is used to regulate the humidity inside the enclosed space by emitting water vapor.

Heating plate: It is used to control the temperature of the grasped objects.

Grasped objects: The objects used in this study are divided into 4 groups. The first group consists of objects made from different materials, including brass, resin, nylon, wood, stainless steel, steel, PTFE, and Al. The second group comprises small balls with varying softness. The third group includes steel blocks with 4 distinct roughness levels: Ra0.8, Ra1.6, Ra3.2, and Ra6.4. The fourth group contains 8 objects with combined properties: “PDMS–hard–rough”, “PDMS–hard–smooth”, “PDMS–soft–rough”, “PDMS–soft–smooth”, “Ecoflex–hard–rough”, “Ecoflex–hard–smooth”, “Ecoflex–soft–rough”, and “Ecoflex–soft–smooth”.

To verify the enhanced effectiveness of the MEG unit in object property recognition, especially in roughness discrimination, the dataset was divided into 2 independent groups. The first group combines the voltage signals of the TENG with the current signals of the MEG, while the second group consists only of the voltage signals of the TENG. The experimental results show that the OPRM performs exceptionally well in recognizing object properties. Specifically, when the model is trained on the dataset that includes MEG current signals, its performance substantially surpasses that of the model trained on the dataset relying solely on voltage signals, with particularly pronounced advantages in the roughness classification task.

In the material classification task, the OPRM model integrated with MEG current signals successfully achieves accurate classification of 8 different materials, with a classification accuracy as high as 99.07%, as shown in Fig. 6B. The receiver operating characteristic curve closely approaches the upper left corner of the coordinate axis, and the area under the curve value reaches 1. This visual representation strongly validates the high accuracy of the model (Fig. S15A). The t-distributed stochastic neighbor embedding method was used to perform dimensionality reduction and visual analysis on the features of the model’s intermediate layer. The results indicate that the boundaries between different object property categories are clearly distinguishable, confirming the model’s excellent performance in feature extraction and classification (Fig. S15B). It is worth noting that even the OPRM model relying solely on voltage signals achieves a high accuracy of 97.92% in the material classification task (Fig. 6C). This achievement can be attributed to the fact that the voltage signals of the triboelectric array provide sufficient material-related features, which is consistent with the analysis results shown in Fig. 4A to D.

In the softness classification task, the OPRM model combined with MEG current signals achieves a perfect recognition accuracy of 100% for 3 levels of softness (Fig. 6D and Fig. S15C and D), while the OPRM model relying solely on voltage signals also shows a high recognition accuracy of 99.26% (Fig. 6E). This phenomenon occurs because the softness of an object can be effectively recognized through the temporal features of a single sensor, and as the number of sensors increases, the recognition accuracy will increase accordingly, although it tends to stabilize after reaching a certain threshold.

However, in the roughness classification task, this study clearly demonstrates that the inclusion of MEG current signals notably improves the recognition accuracy. Specifically, the OPRM model integrated with MEG current signals achieves a recognition accuracy of 95.56% for 4 roughness levels (Fig. 6F and Fig. S15E and F), while the accuracy of the OPRM model relying solely on voltage signals is only 88.89% (Fig. 6G), which is markedly lower than that of the model integrated with current signals. This result highlights the advantage of using the MEG as a force-feedback signal in enhancing the model’s robustness.

For the 8 objects with intertwined properties, the recognition accuracy of the OPRM model combined with MEG current signals is 95.83% (Fig. 6H and Fig. S15G and H). In contrast, the OPRM model relying solely on voltage signals has considerable limitations in classifying these complex objects, with an accuracy of only 90.00% (Fig. 6I). To further demonstrate the superiority of the OPRM in object property recognition, this study also compared the performance of 4 basic classification algorithms (k-Nearest Neighbors, Random Forest, Support Vector Machine, and Long Short-Term Memory) in recognizing the 8 objects with intertwined properties. The experimental results confirm that the proposed OPRM model has more advantages in this 8-classification task (as shown in Fig. S16).

### Demonstration of object property perception system

**Fig. 7.**
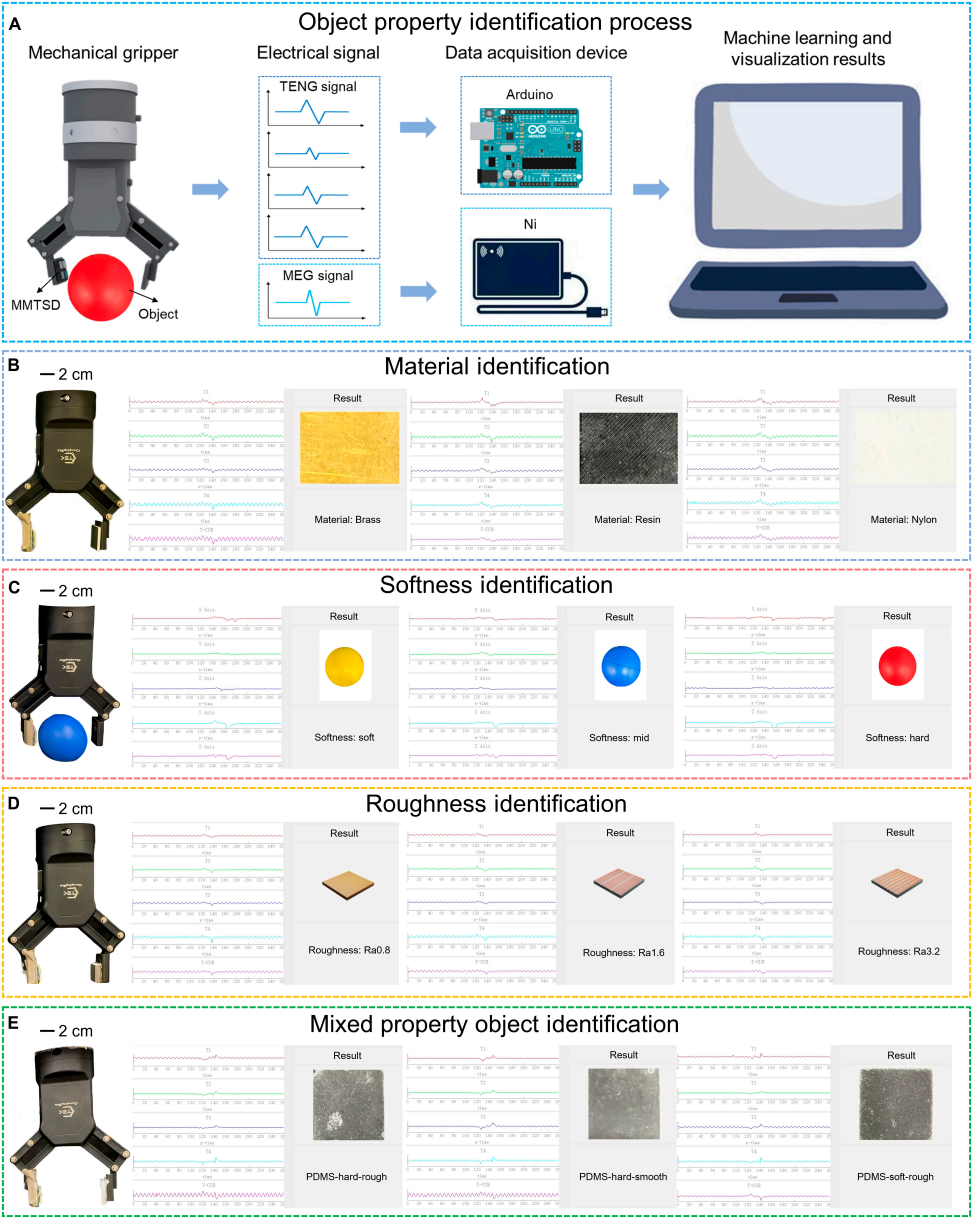
Demonstration of the object property perception system. (A) Process of the object property perception system. (B to D) Identification results of materials, softness, and roughness. (E) Recognition results of mixed property objects.

To validate the efficacy of MMTSD in empowering robots with the capacity to discern the properties of objects in their surroundings, we have devised an integrated system specifically designed for object property perception (as depicted in Fig. 7A). This system comprises pivotal elements: an MMTSD, responsible for accurately capturing detailed information on the surface of objects; a mechanical gripper, functioning as an actuator to achieve precise manipulation and positioning; a microcontroller unit-based module for data processing, acquisition, and transmission, ensuring data handling and instant communication; and a laptop equipped with a customized object property perception interface, serving as the control and display terminal for the system.

During system operation, the mechanical gripper integrated with the MMTSD is pre-programmed to autonomously approach and interact with the target object. Upon contact, the MMTSD rapidly converts object properties into electrical signals. These signals are immediately collected by a data acquisition device consisting of Arduino and Ni acquisition cards. After undergoing thorough processing and integration, the signals are transmitted via a communication channel to the processing system on the laptop.

Within this platform, the OPRM is utilized to conduct an analysis of the acquired data. This analysis enables accurate prediction of the object’s material type and simultaneous visual representation. Leveraging the streamlined network architecture and low-complexity features of the lightweight CNN algorithm, the OPRM can complete a single inference task in approximately 0.1 to 0.4 s, ensuring the system operates stably in real-time scenarios.

The experimental results show that the system can effectively acquire and distinguish the unique waveform signatures of 8 different materials, 3 softness levels, and 4 roughness levels, as illustrated in Fig. 7B and D, Figs. S17 and S18, and Movies S1 to S3. Notably, the system’s prediction accuracy and stability meet the initial expectations. Moreover, the system demonstrates the ability to accurately recognize objects with mixed properties, successfully identifying 3 properties of such objects, as presented in Fig. 7E, Fig. S19, and Movie S4. These findings highlight the versatility and robust reliability of the MMTSD in addressing complex environmental perception challenges.

## Conclusion

This paper introduces an MMTSD by integrating triboelectric and magnetoelastic mechanisms. The MMTSD combines self-power, high precision, and multifunctionality. First, a systematic investigation is conducted to analyze the influence of various factors, including material composition, device size, and surface structure, on TENG performance. At the same time, an in-depth analysis is performed to examine how parameters such as doping concentration, device thickness, and magnetization intensity affect MEG performance. Based on these studies, the optimal structural configuration of the MMTSD is determined. Additionally, this research evaluates a series of performance metrics of the MMTSD, including its frequency response characteristics, pressure-sensing capability, performance under different temperature and humidity conditions, and cyclic stability.

In response to the industry challenge of information decoupling caused by the diversity of object properties, the ambiguity of standards and the mixture of multiple properties, this paper proposes a solution centered around a dual electrical signal sensing mechanism and multi-source signal coding. Specifically, by analyzing the unique charge distribution characteristics of different material surfaces, a distinct “electronic fingerprint” is established for each material. Moreover, a quantitative analysis of object softness is achieved by studying the relationship between the electrical signal response time and the degree of object surface deformation. Meanwhile, voltage–current dual-amplitude signals are used to decouple object surface roughness. Through the decoupling and coding of multi-source signals, an accurate mapping between electrical signals and object properties is successfully established. Notably, by utilizing this dual electrical signal sensing mechanism, the study effectively overcomes the limitation of triboelectric signals being sensitive to external force fluctuations, substantially enhancing the accuracy and robustness of roughness recognition. The results show that the MMTSD outperforms similar sensors in terms of power supply mode, recognition category, and recognition accuracy.

Furthermore, this paper constructs a specialized electrical signal dataset in an open environment and applies machine learning algorithms, which markedly improve the recognition accuracy of object properties. The experimental results show that the recognition accuracy for materials is 99.07%; for softness, it reaches 100%; for roughness, it is 95.56%; and for hybrid properties, it is 95.83%. The finally developed object property perception system, verified through real-world grasping scenarios, demonstrates a high level of adaptability and reliability in complex environments. Meanwhile, this system has excellent portability and can be applied to various intelligent scenarios. For example, in industrial robotics, it can support tasks such as material sorting and defect detection; in humanoid robotics, it can help robots perceive environmental information more accurately and enable more natural human–robot interaction; in medical robotics, it can assist doctors in performing more precise operations. This system is expected to provide strong impetus for the vigorous development of the intelligent robot industry and drive the field of robotics into a new era of increased intelligence, efficiency, and humanization.

## Data Availability

All data needed to evaluate the conclusions in the paper are present in the paper and/or the Supplementary Materials.

## Supplementary Materials

Supplementary 1 Notes S1 to S6 Figs. S1 to S19 Tables S1 to S7 Movies S1 to S4
