# Supplementary material for: Self-Powered Multimodal Tactile Sensing Enabled by Hybrid Triboelectric and Magnetoelastic Mechanisms
Source: Cyborg Bionic Syst. 2025 Jul 2;6:0320. doi: 10.34133/cbsystems.0320 (PMC12214300; doi:10.34133/cbsystems.0320)
Supplement: Supplementary 1 — Notes S1 to S6 Figs. S1 to S19 Tables S1 to S7 Movies S1 to S4 [file cbsystems.0320.f1.zip › Supplementary_Materials_revised.pdf]

Supplementary Materials for

Self-Powered Multimodal Tactile Sensing Enabled by Hybrid Triboelectric and Magnetoelastic Mechanisms

Authors

Example: Xiao Lu[1]†, Tianhong Wang[2]†, Songyi Zhong[2]†, Tianqi Cao[2], Chenghao Zhou[2], Long Li[2], Quan Zhang[2], Shiwei Tian[3], Tao Jin[2]*, Tao Yue[2,4,5]*, Shaorong Xie[1,4,6]*

Affiliations

[1] School of Computer Engineering and Science, Shanghai University, Shanghai 200444, China.

[2] Shanghai Key Laboratory of Intelligent Manufacturing and Robotics, School of Mechatronics Engineering and Automation, Shanghai University, Shanghai 200444, China.

[3] School of Electrical Engineering and Automation, Anhui University. Hefei 230601, China.

[4] Shanghai Institute of Intelligent Science and Technology, Tongji University, Shanghai 200092, China.

[5] School of Future Technology, Shanghai University, Shanghai 200444, China.

[6] Shanghai Key Laboratory of Intelligent Connected Vehicle Cybersecurity, Shanghai 200444, China.

† These authors contributed equally to this work.

*Address correspondence to: robjin@shu.edu.cn (Tao Jin); tao_yue@shu.edu.cn (Tao Yue); Srxie@shu.edu.cn (Shaorong Xie)

This PDF file includes:

Notes S1 to S6
Figs. S1 to S19
Tables S1 to S7
Movies S1 to S4

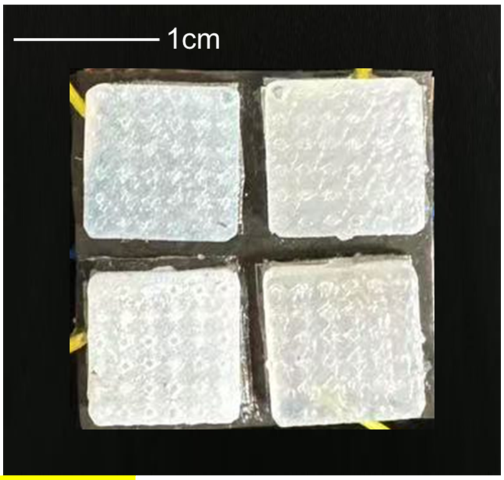

Fig. S1. The optical photo of MMTSD.

Note S1. Structural optimization of MEG

Upon meticulously investigating the pivotal influence of the thickness of FM-CL on its magnetic field intensity, this study prepared FM-CL samples with distinct thicknesses of 1.5 mm, 2 mm, and 2.5 mm. Utilizing a high-precision tensile testing apparatus, within a loading spectrum ranging from 0 to 12N, a systematic analysis was conducted to elucidate the dynamic variations in magnetic flux attributable to the compressive deformation of the material. The definitive outcomes are illustrated in Fig. S2A. The empirical data demonstrates that as the thickness of FM-CL augments, its initial magnetic flux undergoes a marked enhancement, while its response to externally applied forces exhibits an expanded range and heightened linearity. Additionally, the pivotal role of regulating the doping concentration of NdFeB micromagnets in modulating the magnetic field strength of FM-CL post-magnetization has been corroborated. As evident in Fig. S2B, a positive correlation is observed between the output current and the augmentation in micromagnet concentration, consonant with theoretical anticipations. However, it should be noted that as the concentration of micromagnets continues to increase, the compatibility problem with the Ecoflex matrix material becomes prominent, and the polymerization difficulty increases. Considering the current output threshold required in practical applications and the feasibility of material processing, this study selected 85% micromagnet concentration as the optimal production standard. Furthermore, this research delved into the intricacies of how the area of FM-CL influences current output. While maintaining a constant coil turn count and ensuring the coil diameter aligns with the soft magnetic layer, experimental validation established a direct proportional relationship between the FM-CL area and current output. The specific findings are presented in Fig. S2C. Lastly, a thorough analysis was undertaken to examine the effect of coil turn number on the electrical output performance of magnetoelastic sensing (refer to Fig. S2D). The experimental results show that as the number of coil turns increases, the current output of magnetoelastic sensing is significantly increased. The MEG unit parameters used in subsequent experiments are: FM-CL thickness of 2.5mm, area of $2.5 \times 2.5\text{cm}^2$, coil turns of 35, and doping concentration of 85% for micromagnets.

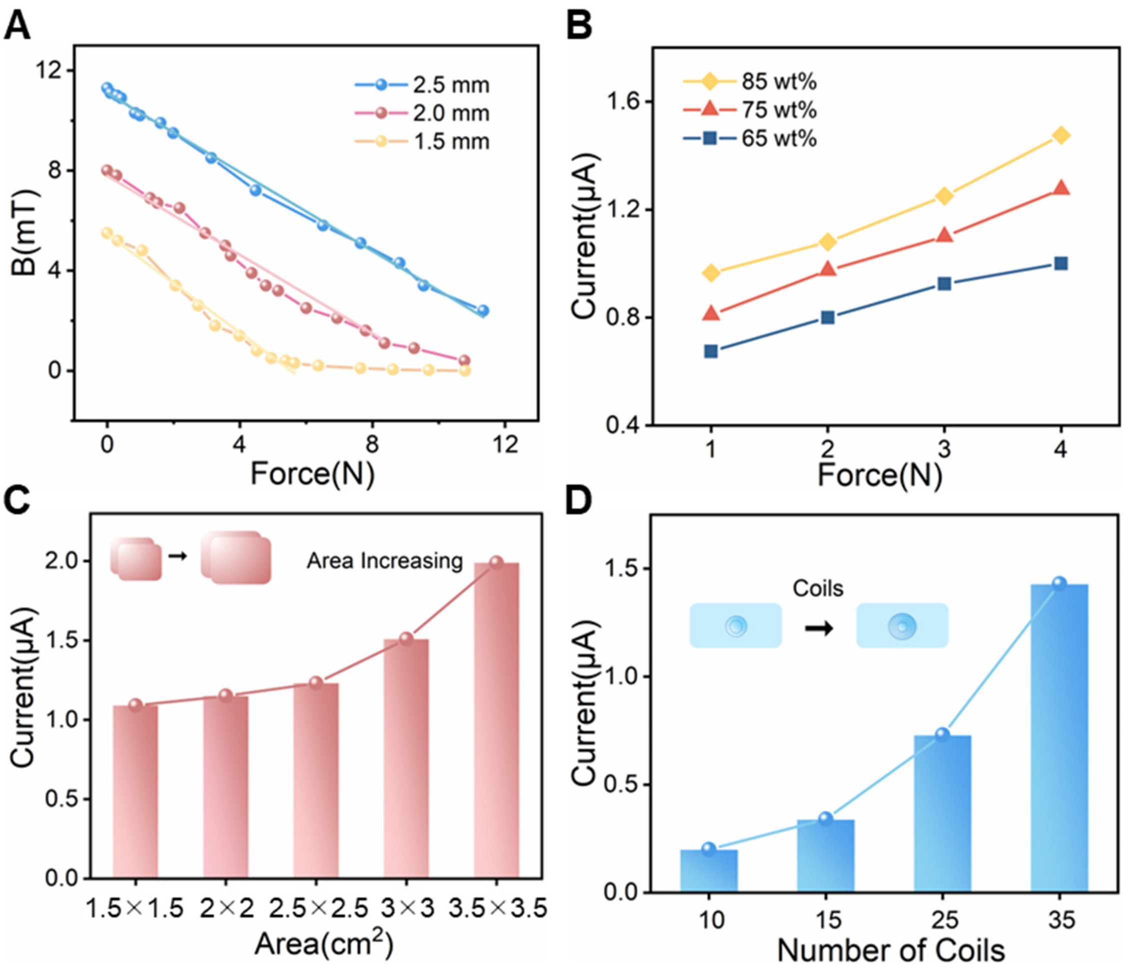

Fig. S2. The influence of structural parameters on MEG performance. (A) The influence of FM-CL thickness on magnetic field strength. (B) The influence of doping concentration of micromagnets on MEG electrical signals. (C) The relationship between the area of FM-CL and MEG electrical signals. (D) The relationship between coil turns and MEG electrical signals.

Note S2. Structural optimization of TENG

When we designed the triboelectric sensing, in order to enhance the triboelectric output performance, a pyramid microstructure was fabricated on the surface of the friction layer. Different proportions of the microstructure on the surface of the friction layer will also affect the electrical output. We fabricated TENG with different proportions of microstructures and tested its performance. As shown in Fig. S3A, it was found that with the increase of the proportion of the microstructure, the voltage first increased and then decreased, reaching the highest when the proportion of the microstructure was 40%. In addition, we tested the relationship between the area of the TENG and the output. As shown in Fig. S3B, the larger the area, the stronger the electrical output. The TENG unit parameters used in subsequent experiments are: area of $1 \times 1 cm^2$, surface structure accounting for 40%. Fig. S3C illustrates the influence of the thickness of the TENG on its output performance. As the thickness increases, the output voltage of the TENG first rises and then decreases. This is because the increase in thickness effectively promotes the generation of charges, resulting in a significant enhancement of the electrical performance. However, with the continuous increase in thickness, the electrostatic induction phenomenon shows a weakening trend, thus having a negative impact on the electrical performance. Fig. S3D demonstrates the influence of the number of branches of the conductive wire on the output performance. The results show that when the number of branches exceeds 3, the voltage of the TENG does not increase significantly, indicating that a number of 3 branches provides sufficient collection capability.

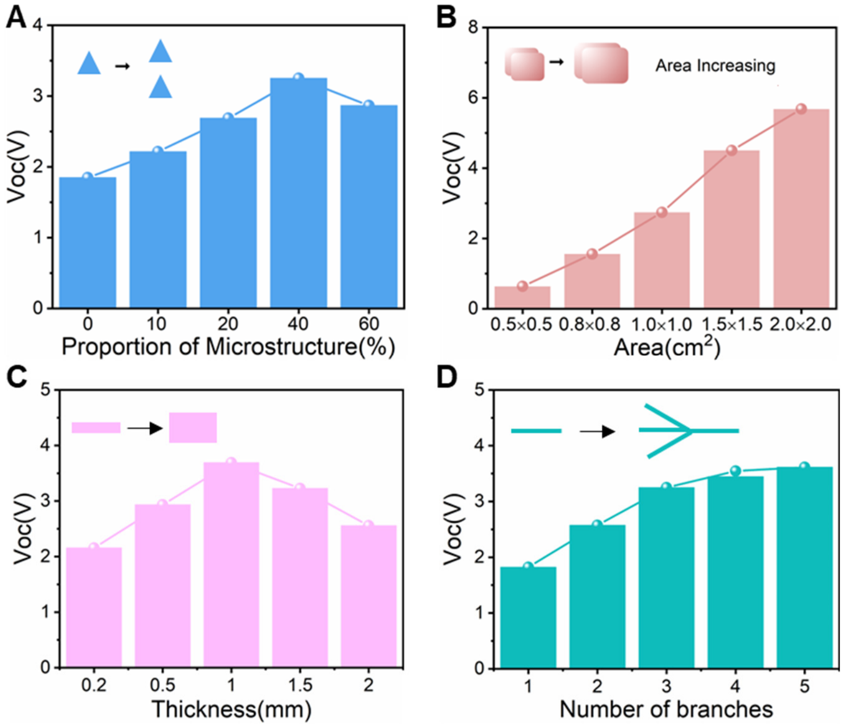

Fig. S3. The influence of structural parameters on TENG performance. (A) The Influence of surface microstructure proportion of friction layer on the electrical properties of TENG. (B) The relationship between friction layer area and TENG electrical performance. (C) The influence of the thickness of the TENG on its performance. (D) The performance of the current collection capability regarding the number of branches of the conductive wire.

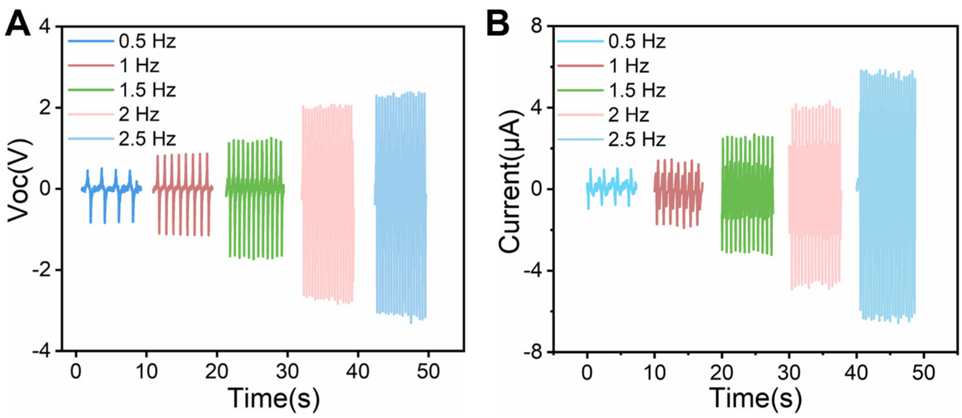

Fig. S4. MMTSD's frequency response waveform. (A) TENG's frequency response voltage waveform and (B) MEG's frequency response current waveform.

Note S3. MMTSD's power supply capability

The power supply capacity of MMTSD depends on impedance matching to obtain the maximum output power. By testing the output performance of MEG sensing and TENG sensing under different external load resistances, it is found that when the load resistance is increased from 1.5MΩ to 1000MΩ, the TENG voltage and current show opposite trends with the change of resistance (Fig. S5A). When the load resistance is 50MΩ, the TENG achieves the maximum output power of 0.22μW (Fig. S5B). The voltage and current of MEG show the same trend as the TENG with the increase of the load (Fig. S5C), but the optimal matching impedance is much smaller than that of the TENG. When the load resistance is 240Ω, the MEG achieves the maximum output power of 51.3μW (Fig. S5D).

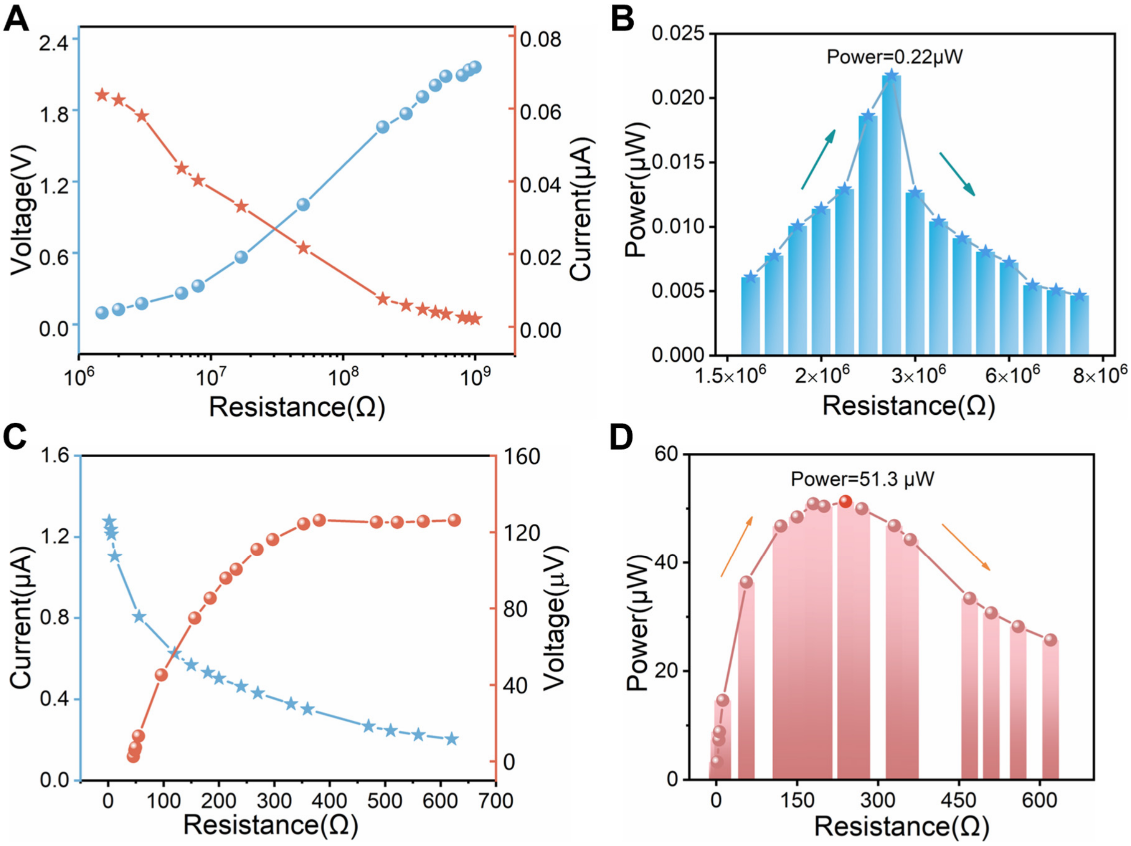

Fig. S5. MMTSD's power supply characteristics. (A to B) Voltage, current, and power response of TENG under different load resistances. (C to D) Voltage, current, and power response of MEG under different load resistances.

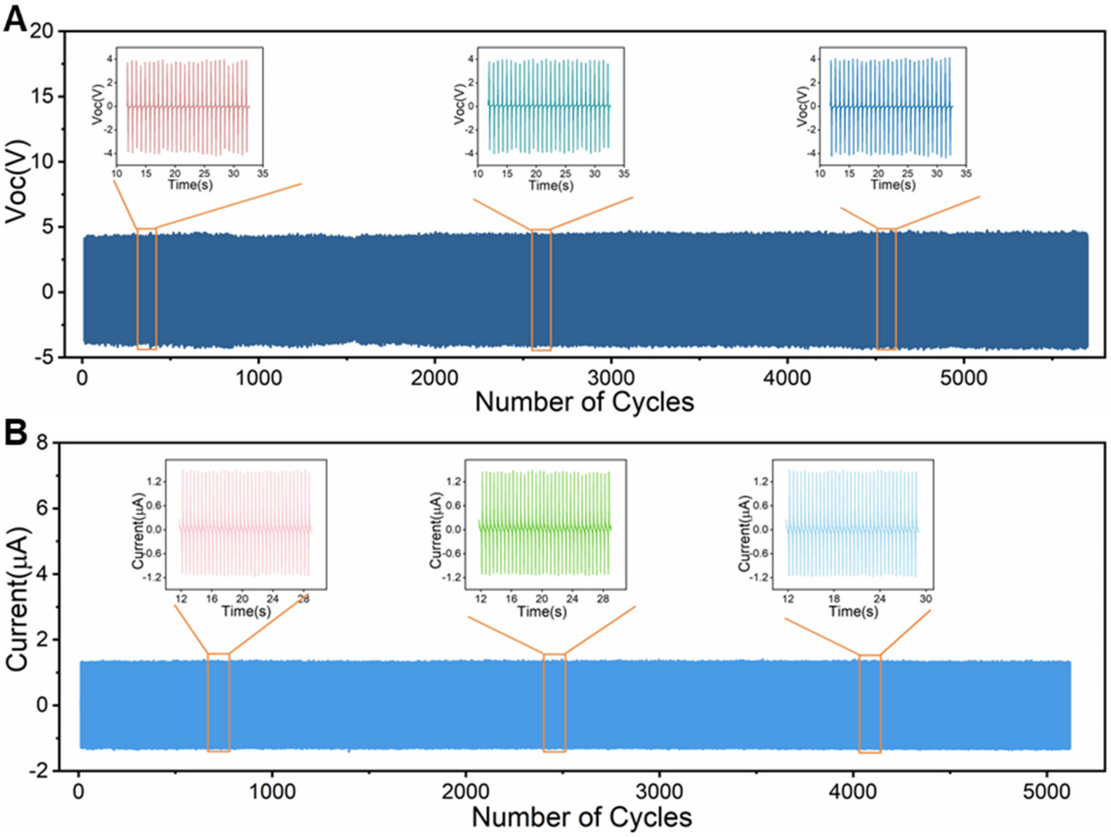

Fig. S6. The cyclic stability of MMTSD. (A) The stability of TENG voltage signal under more than 5000 cycles. (B) The stability of MEG current signal under more than 5000 cycles.

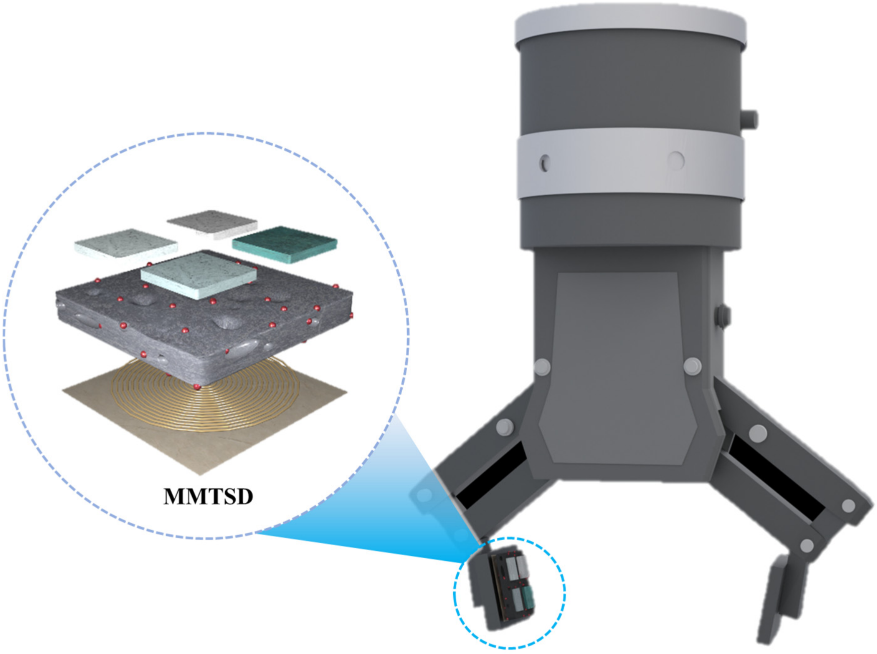

Fig. S7. MMTSD assembly method.

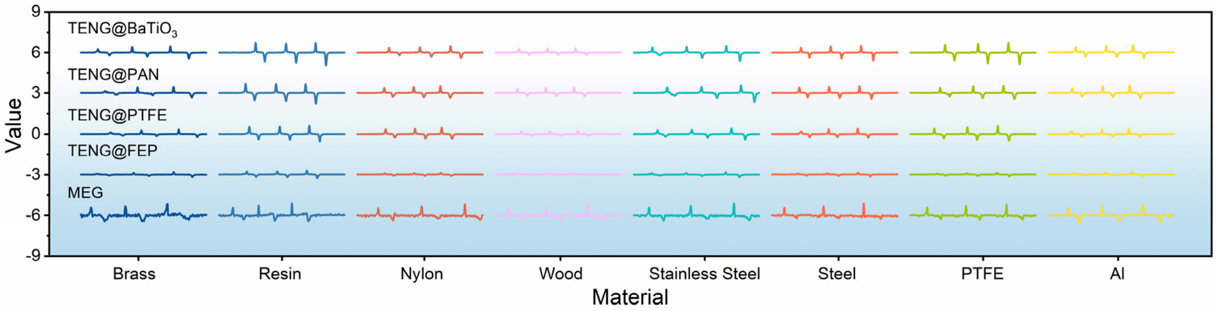

Fig. S8. MMTSD electrical signals of mechanical claws grasping eight different materials of objects.

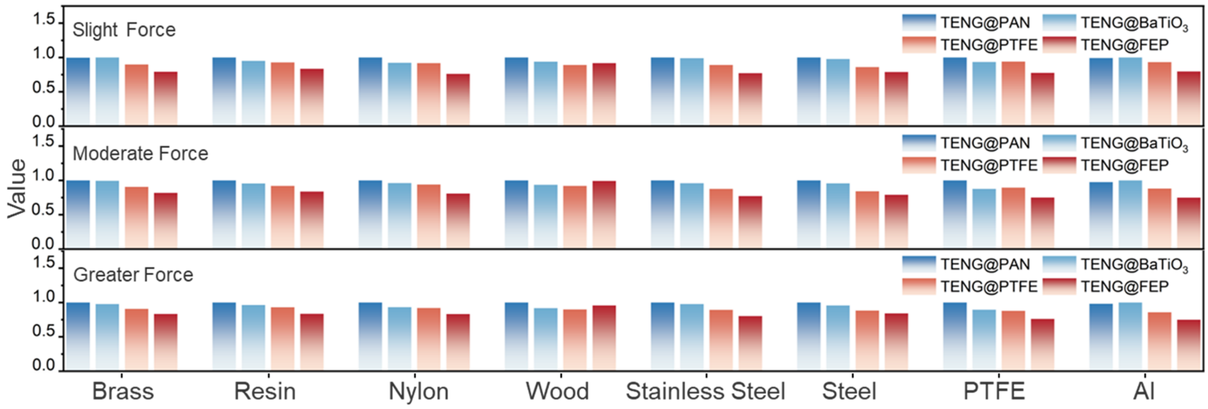

Fig. S9. Normalized data of MMTSD material response voltage peak value.

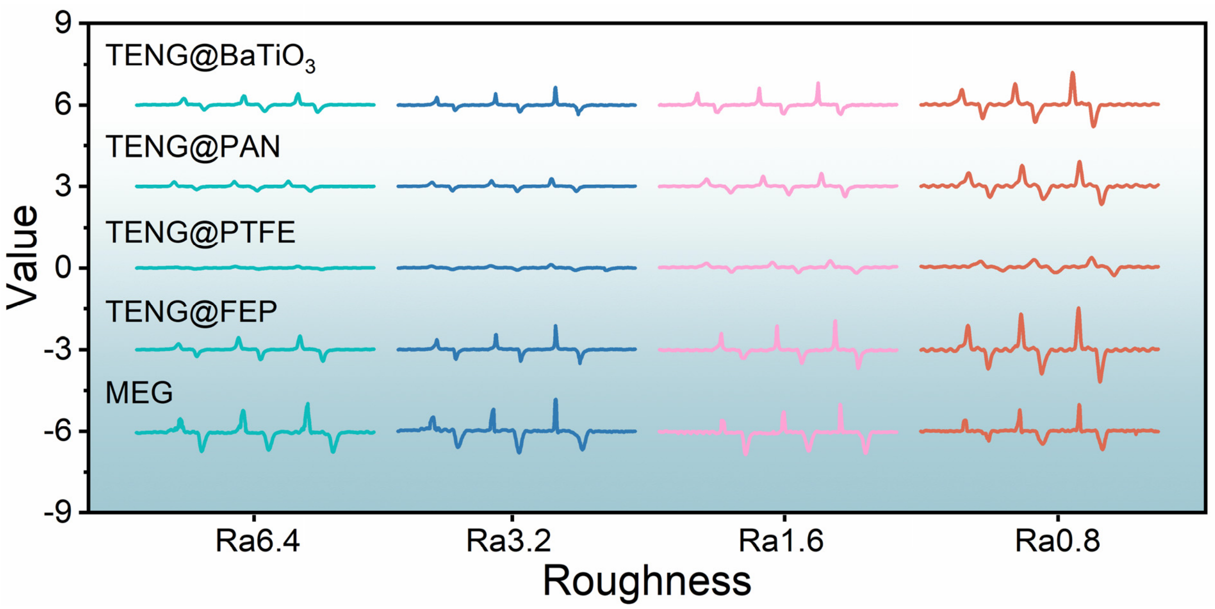

Fig. S10. MMTSD electrical signals of mechanical claws grasping four different roughness objects.

Note S4. Analysis of roughness recognition based on TENG

Relying solely on the triboelectric signal, it is impossible to determine the roughness of an object under the condition of unfixed force, because the force will interfere with the peak signal of the TENG, thus affecting the accuracy of recognition. It can be seen from Fig. S11 that the shapes enclosed by the TENG voltage amplitudes under different roughness and force conditions are the same because the materials are the same. However, the TENG voltage amplitude under a certain force at a certain roughness may overlap with the TENG voltage amplitude under a certain force at another roughness, resulting in misjudgment. Therefore, a stable force feedback unit is required to compensate for the TENG signal.

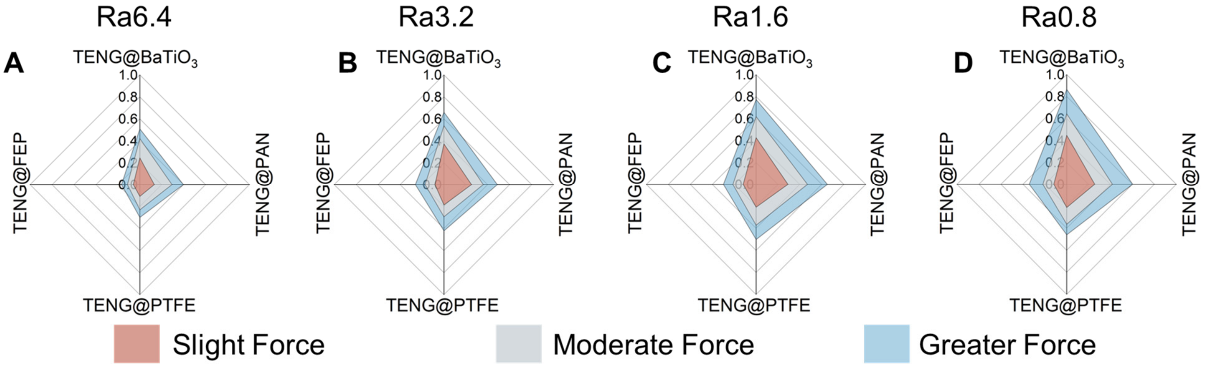

Fig. S11. Analysis of TENG electrical signals under different roughness levels. (A to B) Radar chart of TENG voltage peak corresponding to the increase of surface roughness of steel block from Ra6.4 to Ra0.8.

Note S5. Method for controlling property parameters of objects

To reflect the differences in the three parameters of material, softness, and roughness, we have selected two different types for each parameter, with a total of eight combinations. We chose Ecoflex and PDMS as the materials, and achieved the difference in softness by controlling the thickness of these two materials. We molded objects with different roughness on sandpaper of different mesh sizes. The eight objects with intertwined properties are shown in Fig. S12.

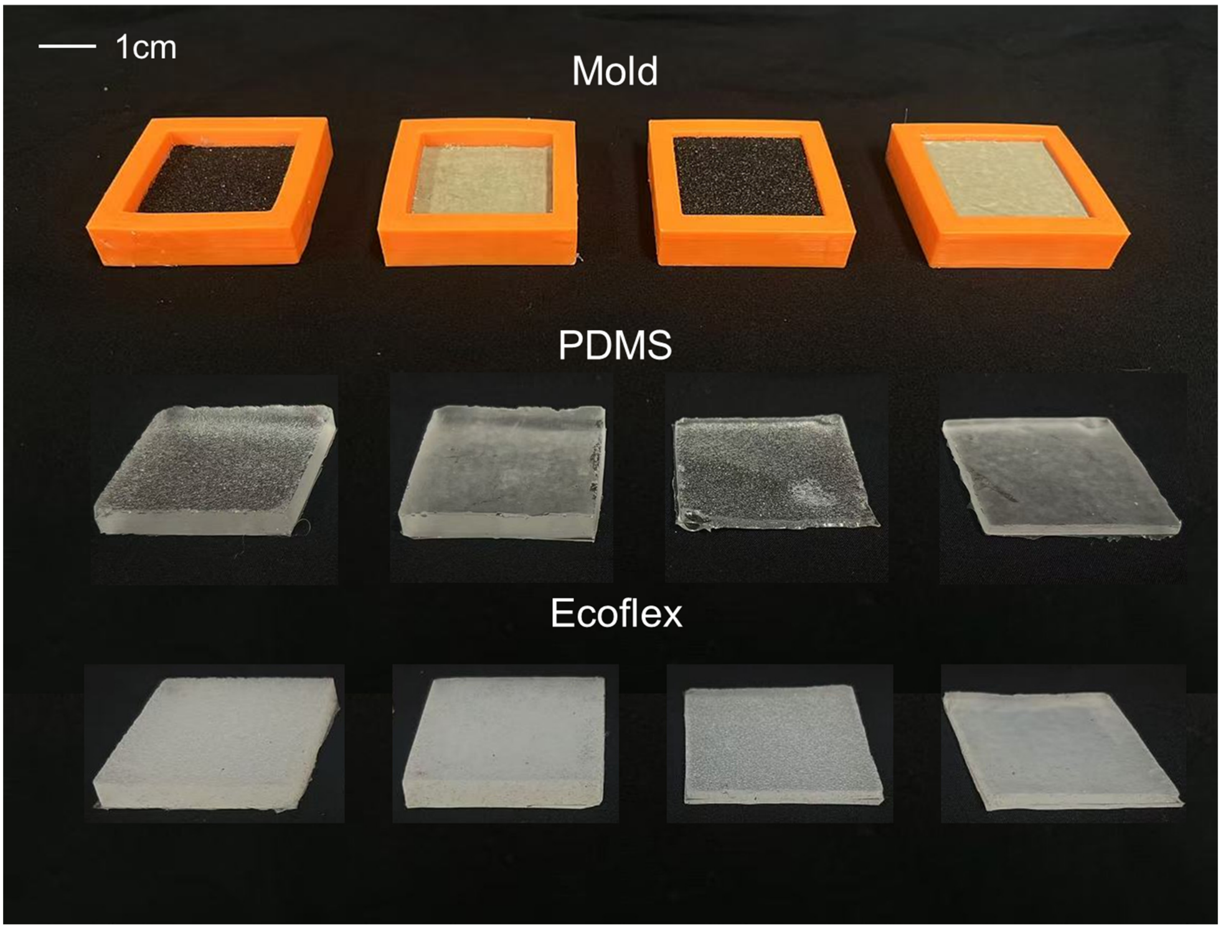

Fig. S12. The eight objects with intertwined properties.

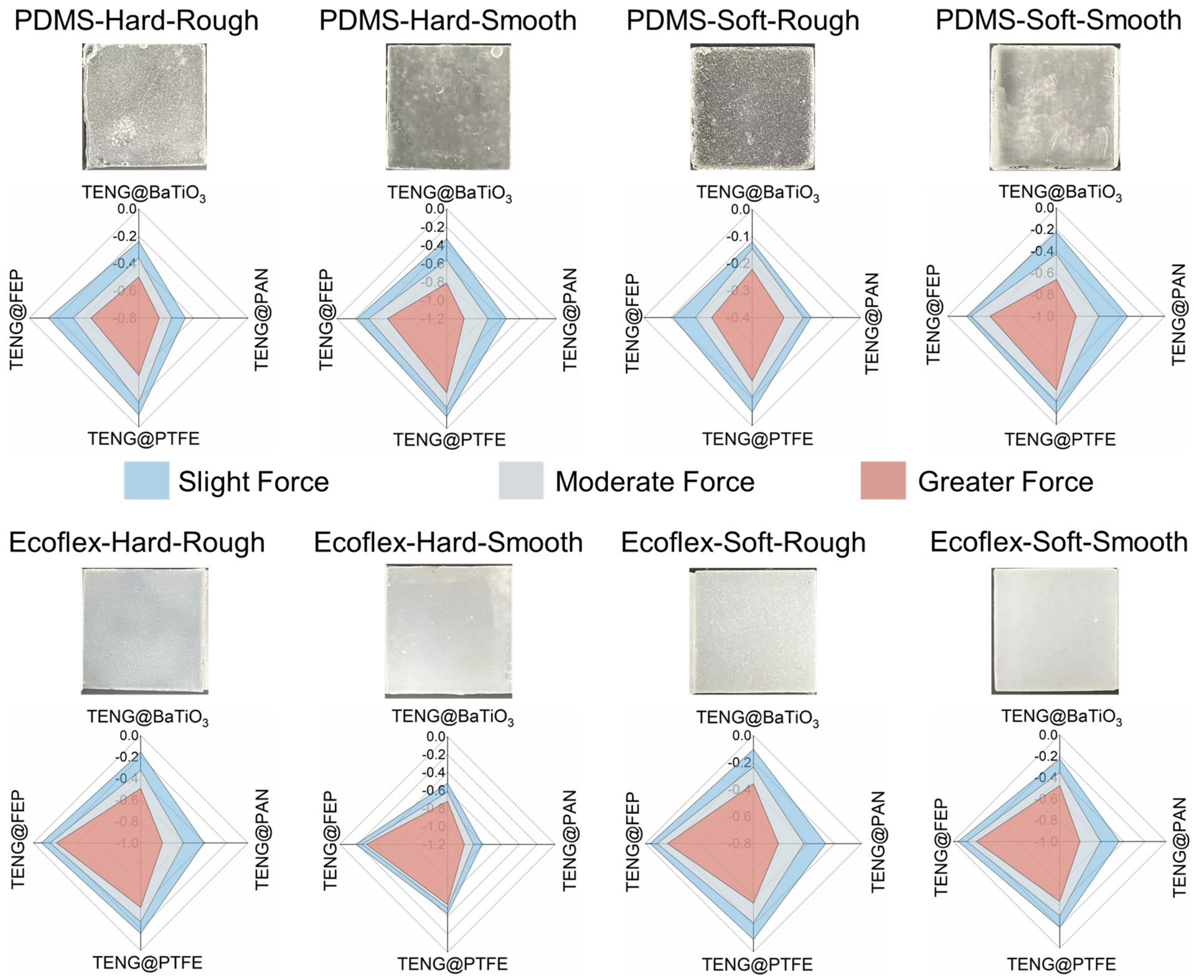

Fig. S13. Radar plot of peak voltage under different contact force conditions.

Note S6. Modeling Framework

The model's construction process encompasses three pivotal stages:

Data Processing: Initially, the electrical signals pertaining to object properties, acquired via MMTSD, undergo preprocessing. This involves filtering out interference and noise through a low-pass filter to enhance signal clarity and the signal-to-noise ratio, thereby laying a solid foundation for model training. Subsequently, voltage and current data are merged, and data slicing techniques are employed to standardize the sample length, ensuring input data consistency. Additionally, data normalization is performed to balance feature scales and bolster the stability and convergence efficiency of model training. During dataset partitioning, we adopt a stratified sampling approach to scientifically allocate the dataset into a training set (70%), a validation set (15%), and a test set (15%), thereby ensuring balanced category distribution and enhancing model generalization.

Deep Feature Extraction: In the feature extraction phase, the CNN's convolutional layer captures the short-term dynamic features and local correlations within the MMTSD electrical signals. Simultaneously, batch normalization stabilizes data distribution, while the pooling layer diminishes feature dimensionality, reduces computational complexity, and effectively mitigates overfitting. Subsequently, a global maximum pooling operation in the temporal dimension generates a fixed-length feature vector, providing an efficacious input for subsequent fully connected layer processing.

Object Property Category Classifier: The classification layer comprises a fully connected layer with 256 neurons, which further refines the features. A Dropout layer is incorporated to prevent overfitting and bolster the model's robustness and generalization capabilities. Ultimately, the output layer, equipped with a softmax activation function, yields the classification result, with the number of neurons corresponding to the number of object property categories.

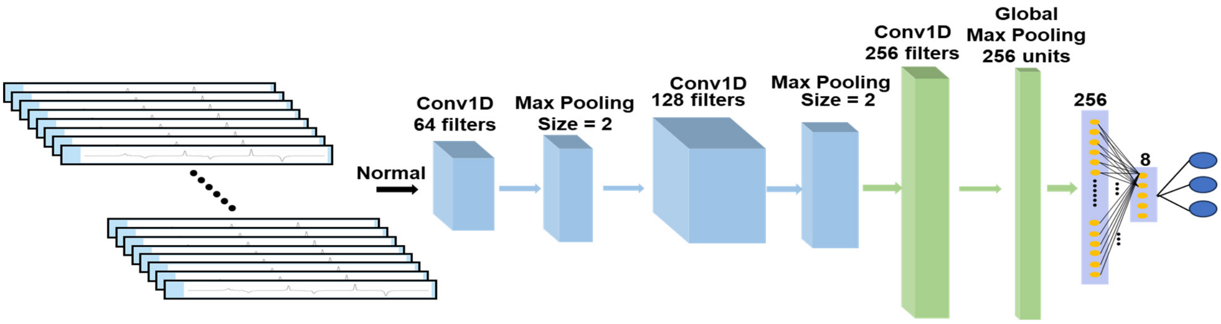

Fig. S14. The model framework for object property recognition.

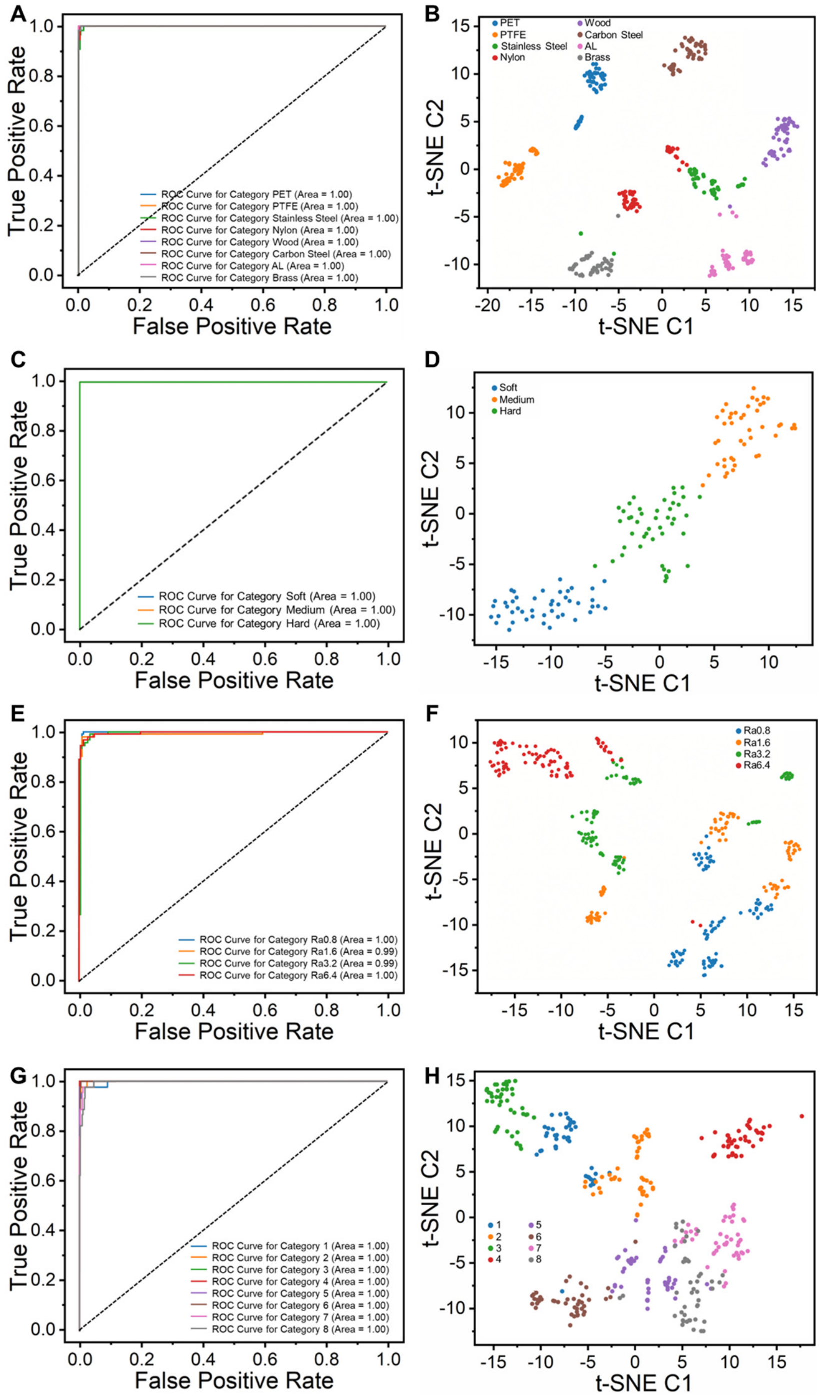

Fig. S15. ROC curve and t-SNE scatter plot in object properties classification task. (A to B) ROC curve and t-SNE scatter plot based on voltage and current fusion dataset in material classification task. (C to D) ROC curve and t-SNE scatter plot based on voltage and current fusion dataset in softness classification task. (E to F) ROC curve and t-SNE scatter plot based on voltage and current fusion dataset in roughness classification tasks. (G to H) ROC curve and t-SNE scatter plot based on voltage and current fusion dataset in eight objects with mixed property classification tasks.

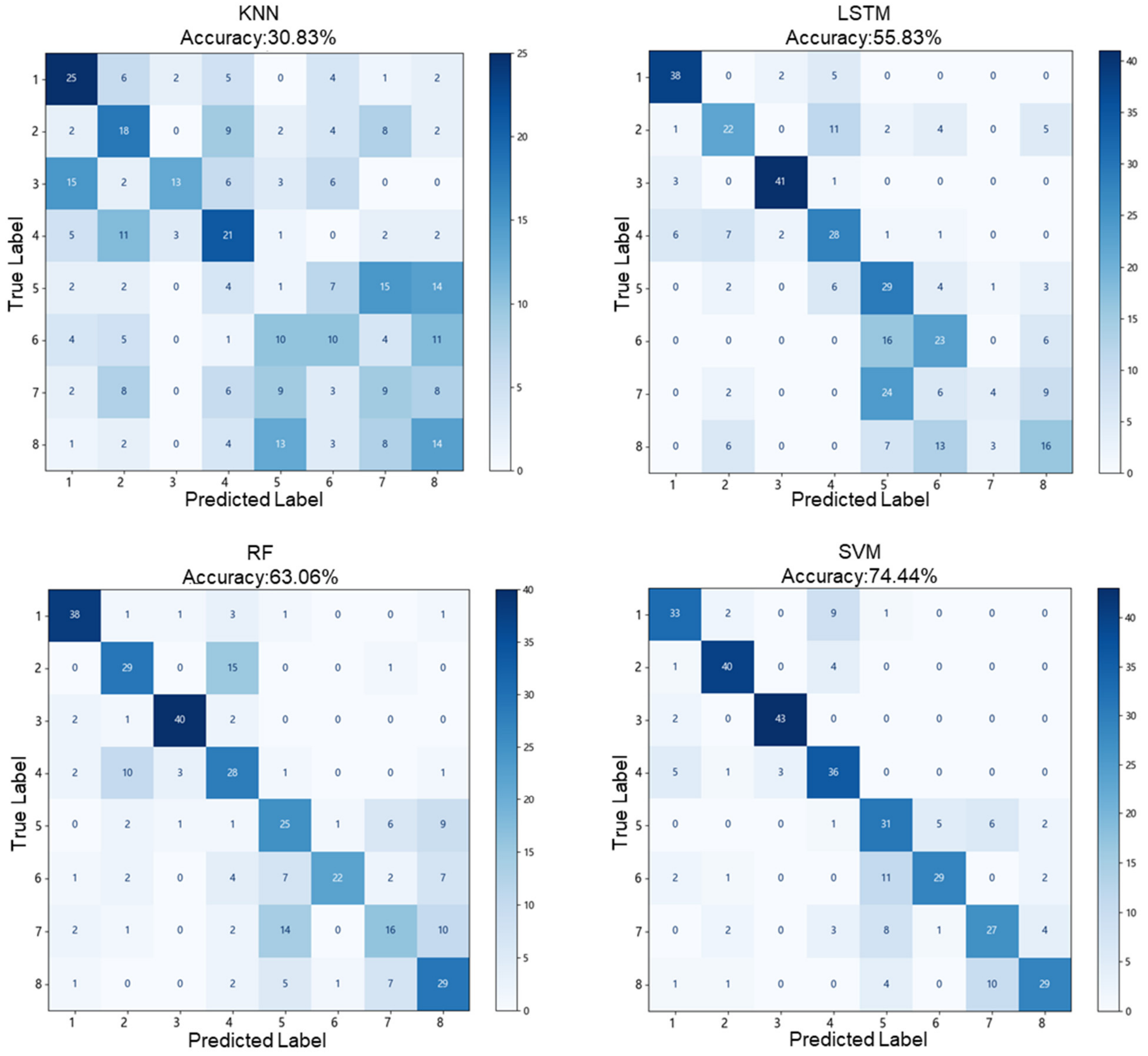

Fig. S16. Comparison of four typical classification algorithms.

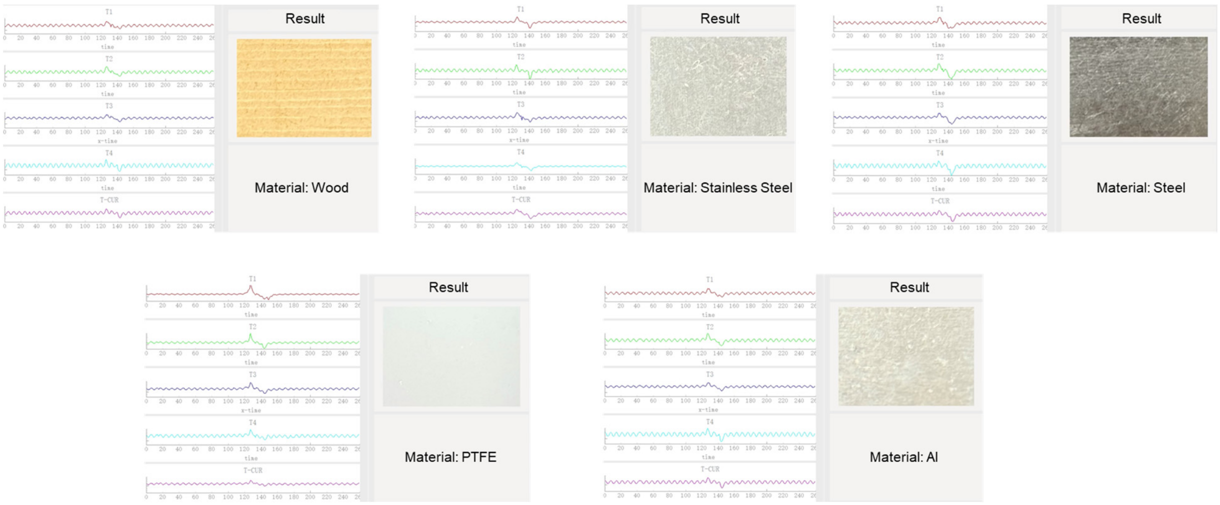

Fig. S17. Identification results of materials.

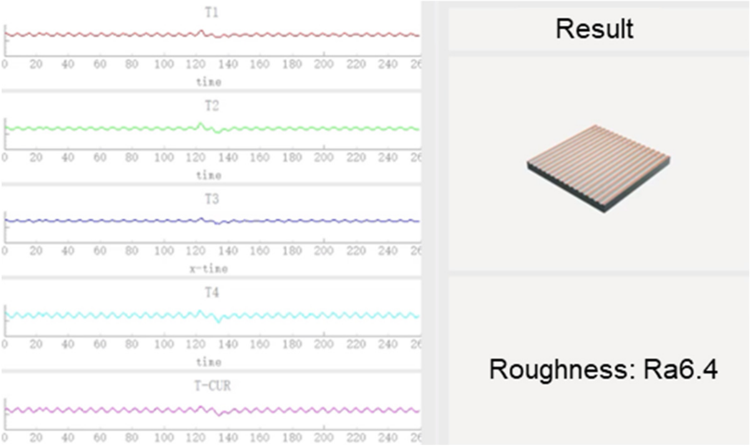

Fig. S18. Identification results of roughness.

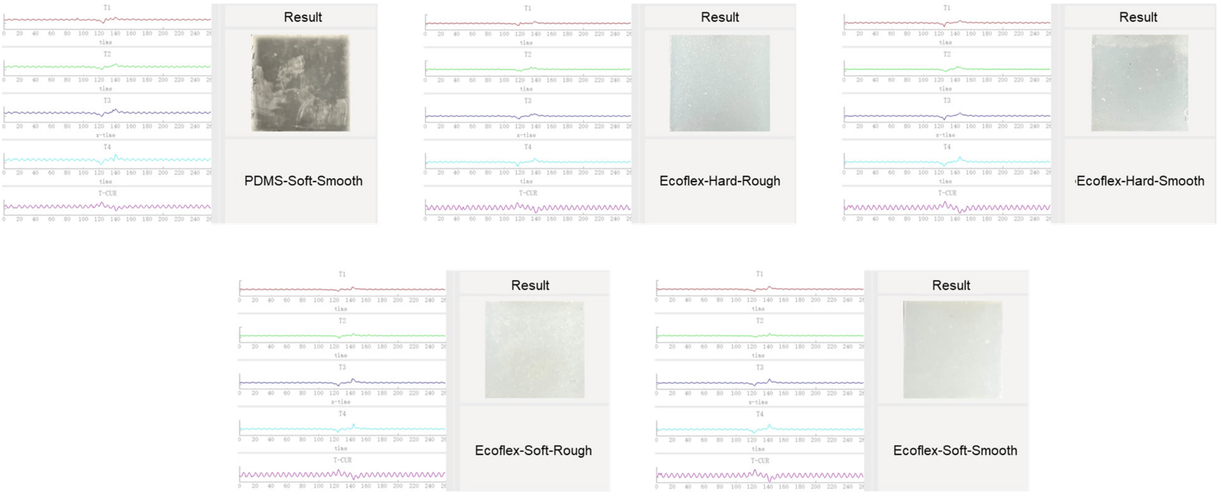

Fig. S19. Recognition results of mixed property objects.

Table S1. Sensitivity and linearity of MMTSD

Parameter	TENG@PAN	TENG@PTFE	TENG@$BaTiO_3$	TENG@FEP	MEG
Sensitivity	0.18V/N	0.14 V/N	0.13 V/N	0.06 V/N	0.42μA/N
R^2	0.991	0.993	0.995	0.992	0.997

Table S2. Standardization data of TENG voltage for eight materials under different forces.

Force	Object	TENG voltage standardization data			
		TENG@PAN	TENG@PTFE	TENG@$BaTiO_3$	TENG@FEP
Slight Force	Brass	1	0.89613	0.99472	0.79225
	Resin	0.95098	0.92861	1	0.83475
	Nylon	0.92118	0.91626	1	0.76026
	Wood	0.93841	0.88979	1	0.91630
	Stainless Steel	0.98854	0.89034	1	0.77087
	Steel	0.97802	0.85871	1	0.78807
	PTFE	0.93333	0.93926	1	0.77481
	Al	1	0.93259	0.9913	0.79480
Moderate Force	Brass	0.99446	0.90590	1	0.81919
	Resin	0.95494	0.91954	1	0.83619
	Nylon	0.96113	0.93993	1	0.80742
	Wood	0.93671	0.92037	1	0.99081
	Stainless Steel	0.95826	0.87646	1	0.77129
	Steel	0.95574	0.84098	1	0.78852
	PTFE	0.87595	0.89410	1	0.75060
	Al	1	0.88072	0.97529	0.74772
Greater Force	Brass	0.97736	0.90755	1	0.83208
	Resin	0.96280	0.92984	1	0.83509
	Nylon	0.93148	0.91852	1	0.83148
	Wood	0.91734	0.89919	1	0.95565
	Stainless Steel	0.97691	0.89343	1	0.80107
	Steel	0.95564	0.88170	1	0.84104
	PTFE	0.89407	0.87796	1	0.76044
	Al	1	0.85782	0.98102	0.74831

Table S3. MMTSD electrical signal parameters (under different softness levels)

Parameter	Softness	TENG@PAN	TENG@PTFE	TENG@BaTiO$_3$	TENG@FEP	MEG
Response Time (s)	Soft	0.6	0.5	0.9	0.7	0.43
	Medium	0.5	0.4	0.6	0.5	0.19
	Hard	0.3	0.3	0.4	0.4	0.12
Voc/Current Peak Value (V/μA)	Soft	0.41	0.17	0.53	0.24	0.58
	Medium	0.50	0.22	0.97	0.35	1.00
	Hard	0.57	0.28	1.08	0.40	1.20

Table S4. The comparison of this work with other research on identifying object properties.

Year	Methods	Recognition Categories	Model	Accuracy	Power Supply Mode	Reference
2025	TENG+MEG	Material, Softness, Roughness	CNN	Material: 0.994 Softness: 1.00 Roughness: 0.956	Self-Power	This Work
2022	TENG array	Material	Linear discriminant analysis algorithm	0.968	Self-Power	[15]
2022	TENG array	Material	VGG	0.962	Self-Power	[14]
2022	Piezoelectric	Multiple objects	Bioinspired olfactory-tactile associated machine-learning strategy	0.969	Self-Power	[22]
2022	Mechanically gated electron channels	Roughness	——	——	External Power Supply	[19]
2023	Capacitive	Softness	——	——	External Power Supply	[21]
2023	Single iontronic slip-sensor	Texture	RF	0.989	External Power Supply	[22]
2024	Piezoresistive array	modulus	DNN	——	External Power Supply	[17]
2020	Piezoresistive + TENG	Multiple objects	SVM	0.981	External Power Supply	[34]
2023	Piezoresistive + TENG	Texture	PCA	——	External Power Supply	[33]
2024	Capacitive + TENG	Multiple objects	CNN	0.984	External Power Supply	[32]
2024	TENG + TENG	Material, Softness	KNN	Material: 0.994 Softness: 1.00	Self-Power	[36]

Table S5. MMTSD electrical signal parameters (under slight force)

	Object	Parameter	TENG@PAN	TENG@PTFE	TENG@BaTiO_3	TENG@FEP	MEG
Slight Force	PDMS-Hard-Rough	Response Time (s)	0.23	0.20	0.33	0.26	0.09
		Voc/Current Peak Value (V/μA)	-0.46	-0.09	-0.24	-0.14	0.64
	PDMS-Hard-Smooth	Response Time (s)	0.44	0.37	0.40	0.30	0.09
		Voc/Current Peak Value (V/μA)	-0.55	-0.13	-0.33	-0.22	0.60
	PDMS-Soft-Rough	Response Time (s)	0.56	0.56	0.56	0.78	0.12
		Voc/Current Peak Value (V/μA)	-0.18	-0.05	-0.12	-0.11	0.53
	PDMS-Soft-Smooth	Response Time (s)	0.56	0.53	0.73	0.50	0.14
		Voc/Current Peak Value (V/μA)	-0.35	-0.10	-0.22	-0.18	0.52
	Ecoflex-Hard-Rough	Response Time (s)	0.27	0.27	0.27	0.23	0.12
		Voc/Current Peak Value (V/μA)	-0.41	-0.16	-0.16	-0.08	0.60
	Ecoflex-Hard-Smooth	Response Time (s)	0.17	0.20	0.20	0.23	0.13
		Voc/Current Peak Value (V/μA)	-0.81	-0.43	-0.52	-0.18	0.59
	Ecoflex-Soft-Rough	Response Time (s)	0.57	0.50	0.54	0.50	0.21
		Voc/Current Peak Value (V/μA)	-0.27	-0.09	-0.10	-0.04	0.47
	Ecoflex-Soft-Smooth	Response Time (s)	0.53	0.56	0.5	0.46	0.22
		Voc/Current Peak Value (V/μA)	-0.44	-0.19	-0.23	-0.05	0.48

Table S6. MMTSD electrical signal parameters (under moderate force)

	Object	Parameter	TENG@PAN	TENG@PTFE	TENG@$BaTiO_3$	TENG@FEP	MEG
**Moderate Force**	PDMS-Hard-Rough	Response Time (s)	0.33	0.20	0.30	0.27	0.08
		Voc/Current Peak Value (V/μA)	-0.56	-0.18	-0.36	-0.32	0.82
	PDMS-Hard-Smooth	Response Time (s)	0.37	0.26	0.33	0.30	0.11
		Voc/Current Peak Value (V/μA)	-0.75	-0.23	-0.56	-0.35	0.85
	PDMS-Soft-Rough	Response Time (s)	0.56	0.87	0.53	0.53	0.14
		Voc/Current Peak Value (V/μA)	-0.21	-0.11	-0.15	-0.19	0.66
	PDMS-Soft-Smooth	Response Time (s)	0.50	0.53	0.46	0.50	0.17
		Voc/Current Peak Value (V/μA)	-0.61	-0.22	-0.43	-0.22	0.66
	Ecoflex-Hard-Rough	Response Time (s)	0.23	0.27	0.23	0.20	0.13
		Voc/Current Peak Value (V/μA)	-0.60	-0.26	-0.32	-0.16	0.79
	Ecoflex-Hard-Smooth	Response Time (s)	0.23	0.20	0.23	0.20	0.13
		Voc/Current Peak Value (V/μA)	-0.91	-0.49	-0.62	-0.25	0.85
	Ecoflex-Soft-Rough	Response Time (s)	0.46	0.43	0.46	0.37	0.15
		Voc/Current Peak Value (V/μA)	-0.43	-0.21	-0.23	-0.09	0.66
	Ecoflex-Soft-Smooth	Response Time (s)	0.53	0.53	0.5	0.40	0.14
		Voc/Current Peak Value (V/μA)	-0.62	-0.30	-0.35	-0.13	0.66

Table S7. MMTSD electrical signal parameters (under greater force)

	Object	Parameter	TENG@PAN	TENG@PTFE	TENG@$BaTiO_3$	TENG@FEP	MEG
**Greater Force**	PDMS-Hard-Rough	Response Time (s)	0.36	0.30	0.37	0.26	0.12
		Voc/Current Peak Value (V/μA)	-0.65	-0.38	-0.50	-0.45	1.23
	PDMS-Hard-Smooth	Response Time (s)	0.40	0.30	0.36	0.23	0.13
		Voc/Current Peak Value (V/μA)	-1.01	-0.39	-0.81	-0.55	1.18
	PDMS-Soft-Rough	Response Time (s)	0.60	0.53	0.63	0.60	0.19
		Voc/Current Peak Value (V/μA)	-0.28	-0.17	-0.22	-0.25	1.03
	PDMS-Soft-Smooth	Response Time (s)	0.46	0.46	0.49	0.46	0.17
		Voc/Current Peak Value (V/μA)	-0.82	-0.32	-0.67	-0.39	1.00
	Ecoflex-Hard-Rough	Response Time (s)	0.27	0.24	0.24	0.24	0.14
		Voc/Current Peak Value (V/μA)	-0.80	-0.40	-0.49	-0.22	1.23
	Ecoflex-Hard-Smooth	Response Time (s)	0.27	0.37	0.24	0.20	0.13
		Voc/Current Peak Value (V/μA)	-1.01	-0.53	-0.72	-0.30	1.19
	Ecoflex-Soft-Rough	Response Time (s)	0.50	0.53	0.50	0.37	0.20
		Voc/Current Peak Value (V/μA)	-0.62	-0.36	-0.36	-0.16	1.01
	Ecoflex-Soft-Smooth	Response Time (s)	0.53	0.70	0.50	0.53	0.2
		Voc/Current Peak Value (V/μA)	-0.81	-0.44	-0.22	-0.13	1.01

Movie S1.
Material identification task.

Movie S2.
Softness identification task.

Movie S3.
Roughness identification task.

Movie S4.
Mixed property object identification task.
